# Supplementary material for: Are we advancing universal health coverage through cataract services? Protocol for a scoping review
Source: BMJ Open. 2020 Jul 8;10(7):e039458. doi: 10.1136/bmjopen-2020-039458 (PMC7348466; doi:10.1136/bmjopen-2020-039458)
Supplement: Supplementary data [file bmjopen-2020-039458supp002.pdf]

Annex 2: Search Results

MEDLINE

1. (cataract$ adj2 surg$ adj2 rate$).tw.
2. (rate$ adj2 cataract$ adj2 surg$).tw.
3. (cataract$ adj2 surg$ adj2 coverage$).tw. 4. (cataract$ adj2 surg$ adj2 outcome$).tw. 5. (incidence adj4 cataract$ adj2 surg$).tw. 6. (rapid adj2 assessment adj3 cataract$).tw. 7. Rapid Assessment of Avoidable Blindness.tw.
8. RAAB.tw.
9. Cataract Extraction/ec [Economics]
10. or/1-9
11. (universal adj2 health adj2 coverag$).tw.
12. cataract$.tw.
13. 11 and 12
14. 10 or 13
15. (cataract$ adj10 (district$ or region$ or province$ or state or states or territor$ or sub-national or national or nation$)).tw.
16. (cataract$ adj10 (country or countries or worldwide or global$ or Asia or China or India or Africa or Europe$)).tw.
17. 15 or 16
18. "Quality of Health Care"/
19. Quality Improvement/
20. Delivery of Health Care/
21. National Health Programs/ 22. State Medicine/
23. Regional Health Planning/ 24. Health Planning/
25. Health Plan Implementation/
26. Health Planning Guidelines/
27. Health Care Reform/
28. Health Resources/
29. Health Priorities/
30. Health Services Research/
31. "health services needs and demand"/
32. Needs Assessment/
33. State Health Plans/
34. Regional Health Planning/
35. Community Health Planning/
36. Hospital Planning/
37. Regional Medical Programs/
38. Health Maintenance Organizations/
39. Comprehensive Health Care/
40. Health Facility Planning/
41. Health Facility Administration/
42. Hospital Administration/
43. exp Hospitals, public/ 44. exp Hospitals, private/
45. health system$.tw.
46. Models, Organizational/
47. Decision Making, Organizational/ 48. Resource Allocation/
49. Efficiency, Organizational/
50. Organizational Innovation/
51. Delivery of Health Care, Integrated/
52. Interdisciplinary Communication/
53. Public Health/
54. Health Promotion/
55. Policy Making/
56. Program Development/
57. Program Evaluation/
58. Quality Control/
59. Quality Assurance, Health Care/
60. Benchmarking/
61. Capacity Building/
62. Health Services Accessibility/ 63. Health Policy/
64. Surgical Procedures, Operative/
65. exp Surgical Equipment/
66. Health Care Rationing/
67. Medically Underserved Area/ 68. Healthcare Disparities/
69. Health Status Disparities/
70. exp Attitude to Health/
71. "Patient Acceptance of Health Care"/
72. Health Education/
73. Public Opinion/

74. Health Behavior/ 77. exp Communication/

75. Social Behavior/ 78. exp Culture/

76. Superstitions/

79. Sex Factors/ 122. quality management.tw.

80. (cataract$ adj3 (woman or women or female or gender or 123. (organisation$ adj3 cultur$).tw.

sex or sexual or equit$ or inequit$)).tw. 124. Disease Management/

81. Women's Rights/ 125. Program Evaluation/

82. Prejudice/ 126. ((provider$ or program$) adj3 (monitor$ or evaluate$ or

83. Vulnerable Populations/ modif$ or practice)).tw.

84. Social Responsibility/ 127. (implement$ adj3 (improve$ or change$ or effort$ or

85. Social Welfare/ issue$ or impede$ or glossary or tool$ or innovation$ or 86. Urban Health Services/ outcome$ or driv$ or examin$ or reexamin$ or scale$ or

87. Rural Health Services/ strateg$ or advis$ or expert$)).tw.

88. Rural Population/ 128. (needs adj3 assess$).tw.

89. Patient Escort Service/ 129. ((education$ or learn$) adj5 (continu$ or material$ or

90. Health Manpower/ meeting or collaborat$)).tw.

91. Health Personnel/ 130. exp Medical audit/

92. Health Workforce/ 131. (audit or feedback or compliance or adherence or

93. Workforce/ training or innovation).ti.

94. human resources for eye health.tw. 132. (guideline$ adj3 (clinical or practice or implement$ or

95. HReH.tw. promot$)).tw.

96. Medical Staff, Hospital/ 133. exp Health Services Accessibility/

97. Nursing Staff, Hospital/ 134. (outreach adj2 (service$ or visit$)).tw.

98. Personnel, Hospital/ 135. (intervention$ adj3 (no or usual or routine or target$ or

99. Professional Competence/ tailor$ or mediat$)).tw. 100. Clinical Competence/ 136. usual care.tw. 101. Medical Errors/ 137. exp Reminder Systems/ 102. Clinical Governance/ 138. remind$.tw.

103. Government Regulation/ 139. (improve$ adj3 (attend$ or visit$ or intervention$ or

104. Public Policy/ adhere$)).tw.

105. Public Health Practice/ 140. (increas$ adj3 (attend$ or visit$ or intervention$ or 106. Public Health Administration/ adhere$)).tw.

107. Health Plan Implementation/ 141. (appointment$ adj3 (miss$ or fail$ or remind$ or follow 108. Public-

Private Sector Partnerships/ up)).tw. 109. Delivery of Health Care, Integrated/ 142. Telephone/ 110.

service delivery.tw. 143. telephone.tw.

111. decision making.tw. 144. Cell Phones/

112. (consensus adj3 (process$ or discuss)).tw. 145. Mobile Applications/

113. stakeholder$.tw. 146. Remote Consultation/

114. Quality Control/ 147. (m-health or e-health or g-health or u-health).tw.

115. Total Quality Management/ 148. (phone$ adj1 (smart or cell)).tw. 116. Quality Indicators, Health Care/ 149. (smartphone$ or cellphone$).tw. 117. Quality Assurance, Health Care/ 150. (hand adj1 held device$).tw.
118. quality assurance.tw. 151. (mobile adj2 (health or healthcare or phone$ or device$ 119. (quality adj2 improv$).tw. or monitor$ or comput$ or app or apps or application)).tw.
120. total quality.tw. 152. Primary Health Care/
121. continuous quality.tw.
153. General Practitioners/ or Physicians, Family/ or Physicians, Primary Care/
154. Primary Prevention/
155. Preventive Health Services/
156. Community Health Services/
157. Community Health Nursing/
158. Health Services, Indigenous/
159. Rural Health Services/ 160. Mobile Health Units/
161. (Ophthalmologist$ or Optometrist$ or Optician$ or Orthopist$ or Refractionists).tw. 162. ((Ophthalmic or eye) adj3 (surgeon$ or nurse$ or technician$ or officer$ or assistant$ or staff$)).tw.
163. Physician's Practice Patterns/
164. Professional Practice/
165. (professional adj3 (practice or develop$ or educat)).tw.
166. Education, Medical, Continuing/
167. exp nurses/
168. Specialties, Nursing/
169. Nurse's Role/ 170. Education, Nursing, Continuing/ 171. (nurse or nurses).tw.
172. ((role or roles) adj3 expan$).tw.
173. (task$ adj3 shift$).tw.
174. exp Medical Records Systems, Computerized/
175. Management Information Systems/
176. Database Management Systems/
177. Computer Systems/ 178. Point-of-Care Systems/
179. Hospital Information Systems/
180. ((health or healthcare) adj4 (record or management system$)).tw.
181. (decision adj5 support).ti. 182. Economics/
183. "costs and cost analysis"/
184. Cost allocation/
185. Cost-benefit analysis/
186. Cost control/
187. Cost savings/
188. Cost of illness/
189. Cost sharing/
190. "deductibles and coinsurance"/ 191. Medical savings accounts/
192. Health care costs/
193. Direct service costs/
194. Drug costs/
195. Employer health costs/ 196. Hospital costs/ 197. Health expenditures/ 198. Capital expenditures/
199. Value of life/
200. exp economics, hospital/ 201. exp economics, medical/ 202. Economics, nursing/
203. Economics, pharmaceutical/
204. exp "fees and charges"/ 205. exp budgets/ 206. (low adj cost).mp. 207. (high adj cost).mp.
208. (health?care adj cost$).mp.
209. (fiscal or funding or financial or finance).tw.
210. (cost adj estimate$).mp.
211. (cost adj variable).mp. 212. (unit adj cost$).mp.
213. (economic$ or pharmacoeconomic$ or price$ or pricing).tw.
214. Uncompensated Care/

215. Reimbursement Mechanisms/ 216. Reimbursement, Incentive/
217. (insurance adj3 (health$ or scheme$)).tw.
218. (financial or economic or pay or payment or copayment or paid or fee or fees or monetary or money or cash or incentiv$ or disincentiv$).tw.
219. ((pay or paying or paid or cost$ or free or wait$ or qualit$) adj3 surg$).tw. 220. (will$ adj3 pay$).tw. 221. (waiting adj2 time).tw.
222. ((surgery or surgical or surgeon$ or ophthalmologist$) adj2 (experience$ or supervis$ or rate or rates or output or volume or uptake number$ or coverage or annual$)).tw.
223. Gross Domestic Product/
224. Medicare/
225. human development index.tw.
226. gross domestic product.tw.
227. (HDI or GDP).tw.
228. Cataract Extraction/sn [Statistics & Numerical Data] 229. (global adj2 burden adj2 cataract$).tw.
230. (cataract$ adj3 (cost$ or income$ or price$ or reimburse$)).tw.
231. exp Patient Acceptance of health Care/
232. exp Attitude to Health/ 233. exp Health Behavior/ 234. Health Education/
235. exp Patient Education as Topic/
236. exp Health Promotion/ 237. Socioeconomic Factors/
238. exp Poverty/ 239. Social Class/
240. Educational Status/
241. ((school or education$) adj3 (status or level$ or attain$ or achieve$)).tw. 242. Employment/
243. Healthcare Disparities/
244. Health Status Disparities/ 245. exp Medically Underserved Area/
246. Rural Population/
247. Urban Population/ 248. exp Ethnic Groups/ 249. Minority Groups/
250. Vulnerable Populations/
251. ((health$ or social$ or racial$ or ethnic$) adj5 (inequalit$ or inequit$ or disparit$ or equit$ or disadvantage$ or depriv$)).tw.
252. (disadvant$ or marginali$ or underserved or under served or impoverish$ or minorit$ or racial$ or ethnic$).tw.
253. or/18-252 254. 17 and 253 255. 14 or 254
256. (femtosecond or phaco$ or keratometry or vitrectomy or endophthalmitis).ti.
257. (glaucoma$ or intraocular or IOL or keratoplast$ or refractive or retinopathy or tear or uveitis).ti.
258. or/256-257 259. 255 not 258
260. limit 259 to yr="2010 -Current"
