# Supplementary material for: Are we advancing universal health coverage through cataract services? Protocol for a scoping review
Source: BMJ Open. 2020 Jul 8;10(7):e039458. doi: 10.1136/bmjopen-2020-039458 (PMC7348466; doi:10.1136/bmjopen-2020-039458)
Supplement: Author's manuscript [file bmjopen-2020-039458.draft_revisions.pdf]

BMJ Open

BMJ Open is committed to open peer review. As part of this commitment we make the peer review history of every article we publish publicly available.

When an article is published we post the peer reviewers' comments and the authors' responses online. We also post the versions of the paper that were used during peer review. These are the versions that the peer review comments apply to.

The versions of the paper that follow are the versions that were submitted during the peer review process. They are not the versions of record or the final published versions. They should not be cited or distributed as the published version of this manuscript.

BMJ Open is an open access journal and the full, final, typeset and author-corrected version of record of the manuscript is available on our site with no access controls, subscription charges or pay-per-view fees (http://bmjopen.bmj.com).

If you have any questions on BMJ Open's open peer review process please email info.bmjopen@bmj.com

ARE WE ADVANCING UNIVERSAL HEALTH COVERAGE THROUGH CATARACT SERVICES? PROTOCOL FOR A SCOPING REVIEW

Journal:	*BMJ Open*
Manuscript ID	bmjopen-2020-039458
Article Type:	Protocol
Date Submitted by the Author:	16-Apr-2020
Complete List of Authors:	Lee, Chan; London School of Hygiene and Tropical Medicine, International Centre for Eye Health<br>Ramke, Jacqueline; LSHTM,<br>McCormick, Ian; London School of Hygiene and Tropical Medicine, Department of Clinical Research<br>Zhang, Justine; London School of Hygiene and Tropical Medicine Faculty of Infectious and Tropical Diseases, International Centre for Eye Health, Clinical Research Department<br>Aghaji, Ada; London Sch Hyg ,<br>Mwangi, Nyawira; London School of Hygiene and Tropical Medicine, ; Kenya Medical Training College,<br>Burn, Helen; Brighton and Sussex University Hospitals NHS Trust<br>Gordon, Iris<br>Yusufu, Mayinuer; Beijing Tongren Hospital; Beijing Institute of Ophthalmology<br>He, Mingguang; Sun Yat-sen University, State Key Laboratory of Ophthalmology, Zhongshan Ophthalmic Center; University of Melbourne, Centre for Eye Research Australia, Royal Victorian Eye and Ear Hospital<br>Silva, Juan Carlos; Pan American Health Organization, Life Course ; PAHO<br>Burton, Matthew J; London School of Hygiene and Tropical Medicine
Keywords:	Cataract and refractive surgery < OPHTHALMOLOGY, EPIDEMIOLOGY, PUBLIC HEALTH, Adult surgery < SURGERY, Ophthalmology < SURGERY

SCHOLARONE™ Manuscripts

I, the Submitting Author has the right to grant and does grant on behalf of all authors of the Work (as defined in the below author licence), an exclusive licence and/or a non-exclusive licence for contributions from authors who are: i) UK Crown employees; ii) where BMJ has agreed a CC-BY licence shall apply, and/or iii) in accordance with the terms applicable for US Federal Government officers or employees acting as part of their official duties; on a worldwide, perpetual, irrevocable, royalty-free basis to BMJ Publishing Group Ltd ("BMJ") its licensees and where the relevant Journal is co-owned by BMJ to the co-owners of the Journal, to publish the Work in this journal and any other BMJ products and to exploit all rights, as set out in our licence.

The Submitting Author accepts and understands that any supply made under these terms is made by BMJ to the Submitting Author unless you are acting as an employee on behalf of your employer or a postgraduate student of an affiliated institution which is paying any applicable article publishing charge ("APC") for Open Access articles. Where the Submitting Author wishes to make the Work available on an Open Access basis (and intends to pay the relevant APC), the terms of reuse of such Open Access shall be governed by a Creative Commons licence – details of these licences and which Creative Commons licence will apply to this Work are set out in our licence referred to above.

Other than as permitted in any relevant BMJ Author's Self Archiving Policies, I confirm this Work has not been accepted for publication elsewhere, is not being considered for publication elsewhere and does not duplicate material already published. I confirm all authors consent to publication of this Work and authorise the granting of this licence.

For peer review only

ARE WE ADVANCING UNIVERSAL HEALTH COVERAGE THROUGH CATARACT SERVICES? PROTOCOL FOR A SCOPING REVIEW

Authors:

Chan Ning Lee[1,2], Jacqueline Ramke[1,3], Ian McCormick[1], Justine H. Zhang[1,4], Ada Aghaji [1,5], Nyawira Mwangi[1,6], Helen Burn[1,7], Iris Gordon[1], Mayinuer Yusufu[8], Mingguang He[9], Juan Carlos Silva[10], Matthew J Burton[1,11]

Affiliations

1. International Centre for Eye Health, London School of Hygiene and Tropical Medicine, London, United Kingdom
2. St Paul's Eye Unit, Royal Liverpool University Hospital, Liverpool, United Kingdom
3. School of Optometry and Vision Science, University of Auckland, Auckland, New Zealand
4. Manchester Royal Eye Hospital, Manchester, United Kingdom
5. Department of Opthalmology, College of Medicine, University of Nigeria, Enugu, Nigeria
6. Department of Clinical Medicine, Kenya Medical Training College, Nairobi, Kenya
7. Beijing Institute of Ophthalmology, Beijing Tongren Eye Center, Beijing Tongren Hospital, Capital Medical University, Beijing, China
8. Department of Ophthalmology, Stoke Mandeville Hospital, Aylesbury, United Kingdom
9. Centre for Eye Research Australia, University of Melbourne, Royal Victorian Eye and Ear Hospital, East Melbourne, Australia
10. Division of Blindness Prevention, Pan American Health Organization, Bogota, Colombia
11. Moorfields Eye Hospital, London, United Kingdom

For peer review only - http://bmjopen.bmj.com/site/about/guidelines.xhtml

ABSTRACT

Introduction

Universal Health Coverage (UHC) includes the dimensions of equity in access, quality services that improve health, and protection against financial hardship. Cataract continues to be the leading cause of blindness globally, despite cataract surgery being an efficacious intervention. The aim of this scoping review is to map the nature, extent and global distribution of data on cataract services for UHC in terms of equity, access, quality and financial protection.

Methods and analysis

The search will be constructed by an Information Specialist and undertaken in MEDLINE, Embase and Global Health databases. We will include all published non-interventional primary research studies and systematic reviews that report a quantitative assessment of access, equity, quality or financial protection of cataract surgical services for adults at the sub-national, national, regional, or global level from population-based surveys or routinely collected health service data since 1 January 2000.

Screening and data charting will be undertaken using Covidence systematic review software. Titles and abstracts of identified studies will be screened by two authors independently. Full text articles of potentially relevant studies will be obtained and reviewed independently by two authors against the inclusion criteria. Any discrepancies between the authors will be resolved by discussion, with a third author as necessary. A data charting form will be developed and piloted on three studies by three authors, and amendments made as necessary. Data will be extracted by two reviewers independently and summarised narratively and using maps.

Ethics and dissemination

Ethical approval was not sought as the scoping review will only use published and publicly accessible data. The review will be published in an open access peer-reviewed journal. A summary of the results will be developed for website posting, stakeholder meetings, and inclusion in the ongoing *Lancet Global Health* Commission on Global Eye Health.

For peer review only

For peer review only - http://bmjopen.bmj.com/site/about/guidelines.xhtml

STRENGTHS AND LIMITATIONS

- The broad scope of this review will result in the first synthesis to date of data on the UHC dimensions of cataract surgical services.
- Another strength is that we will include studies from all world regions and high-, low- and middle-income countries with no language restrictions, to give a global picture of cataract services.
- A potential limitation is the paucity of available information on the 'financial protection' dimension.

For peer review only

For peer review only - http://bmjopen.bmj.com/site/about/guidelines.xhtml

INTRODUCTION

Eye health and vision impairment represent a major global health concern. In the recent *World Report on Vision,* the World Health Organization (WHO) outlined how the provision of quality eye care services contributes directly to achieving Universal Health Coverage (UHC).[1] WHO estimated that in 2020 up to 2.2 billion people have some form of vision impairment and that this figure is projected to rise leading to an increased burden on health systems.[1]

Monitoring progress towards UHC

The WHO defines UHC in the following terms:

"Universal health coverage (UHC) means that all people and communities can use the promotive, preventive, curative, rehabilitative and palliative health services they need, of sufficient quality to be effective, while also ensuring that the use of these services does not expose the user to financial hardship".[2]

It has three broad principles: (1) equity in access; (2) quality services that improve health; (3) protection against financial risk. The central choices involved in UHC are often illustrated using a cube (Figure 1). The size of the inner cube indicates the available funding, reflecting budget constraints. Policy makers have hard decisions to make in order to maximise the public health benefit in relation to (1) who is covered, (2) which services are covered, and (3) the proportion of the direct costs covered.

Figure 1: The three dimensions to consider when moving towards universal health coverage (from WHO[3]).

For peer review only

WHO and the World Bank have developed a framework for tracking progress towards UHC.[4] This focuses on two elements:

1. measuring the coverage of essential health services, as a proportion of the population that can access essential quality health services; and
2. measuring financial protection by determining the proportion of the population in whom direct payment made to obtain health services leads to financial hardship and/or a threat to living standards.[4]

A variety of different types of indicators are used. *Effective service coverage indicators* measure the proportion of people in need of services who receive services of sufficient quality to obtain potential health gains; these are preferred if available. *Service coverage indicators* measure the proportion of the population that needs a service that receive it. *Proxy indicators* are sometimes used where service coverage indicators are not available, but provide a correlated indication of the provision of a health service.[4 5]

The WHO and World Bank have selected a panel of 16 "tracer indicators" to monitor progress towards UHC.[2] There is currently no eye health related indicator among this panel though *effective cataract surgical coverage* and *effective refractive error coverage* were proposed in WHO's Thirteenth General Programme of Work 2019 – 2023 Impact Framework.[6] In addition to measuring population level coverage, it is very important to measure equity in service provision, by disaggregating the data and comparing sub-populations such as wealth quintiles, education, sex, age and geographical region.[4]

Monitoring cataract services for UHC

Cataract is the leading cause of blindness globally and is the second leading cause of vision impairment.[7] The last three decades have seen a marked increase in available data on vision impairment due to cataract, as well as cataract services. These data have enabled calculation of indicators of *access* and *quality* of cataract surgery, including:

- Cataract Surgical Rate (CSR): the number of cataract operations per million population per year.[8]
- Cataract Surgical Coverage (CSC): the number of people in a population who have received cataract surgery as a proportion of those having operable and operated cataract.[6]
- Cataract Surgical Outcome (CSO): the presenting visual acuity of the operated eye.
- Effective cataract surgical coverage (eCSC): the number of people in a population with operated cataract and a visual acuity of 6/18 or better as a proportion of those having operable and operated cataract.[9]

Effective Cataract Surgical Coverage (eCSC), has the characteristics of an *Effective service coverage indicator,* as preferred by the WHO/World Bank, as it combines information on the proportion of the population covered and the outcome of the surgical intervention.[10 11]

Disaggregation of larger datasets has allowed analyses of *equity* in cataract surgery as well, for example in highlighting existing gender disparities in CSC.[12-16] Much less data are available quantifying financial aspects of cataract services. To our knowledge, no existing synthesis of the distribution and quantity of known evidence for the UHC dimensions of cataract surgery has been undertaken. The aim

For peer review only - http://bmjopen.bmj.com/site/about/guidelines.xhtml

of this scoping review is to map the nature, extent and global distribution of data on cataract surgical services for UHC in terms of equity, access, quality and financial protection.

We chose to undertake a scoping review rather than an alternative evidence synthesis approach because we wished to identify and map the available evidence, which we anticipate will be heterogeneous.[17]

METHODS

Objectives / Scoping review questions

We aim to answer the following two questions in relation to cataract services for UHC:

1. What is the nature, extent and global distribution of data on the coverage and effectiveness of cataract services?
2. What is the nature, extent and global distribution of data on financial protection in relation to cataract services?

Protocol and registration

The protocol for this scoping review is reported according to the relevant sections of the PRISMA Extension for Scoping Reviews (PRISMA-ScR) guideline (Annex 2).[18] The protocol is registered on the Open Science Framework (https://osf.io/k3mwg/).

Eligibility Criteria

We will include all published prospective and retrospective primary research studies and systematic reviews that report a quantitative assessment of access, equity, quality or financial protection of cataract surgical services for adults at the sub-national, national, regional, or global level (examples outlined in Table 1). We will include population-level observational studies and reports, including those that use routinely collected data (such as in health information systems) and household surveys such as Rapid Assessment of Avoidable Blindness (RAAB) surveys. We will exclude intervention studies and studies within clinical sub-populations as their outcomes can be different to the general population (e.g. people with diabetes, people with age related macular degeneration). We will exclude studies focused exclusively on cataract services for children (aged under 18 years), as these services differ substantially from those for age-related cataract.

We acknowledge that there are many quantitative and qualitative elements of health care *quality* as defined by the WHO.[19] However, for the purposes of this review we will focus on only two—vision outcomes of cataract surgery and reported complications. We anticipate the literature reporting

For peer review only - http://bmjopen.bmj.com/site/about/guidelines.xhtml

financial protection associated with cataract surgery will be limited. We will include studies and surveys that report 'catastrophic' and 'impoverishing' spending on cataract surgery according to WHO definitions, as well as other related measures of personal and government expenditure on cataract surgery and service provision (Table 1).

Studies will be limited to those including data collected since 1 January 2000 to provide a contemporary view of cataract services. The search strategy will be undertaken without language restrictions and translation will be arranged when required.

Table 1: Primary and secondary outcomes included in the review, mapped against UHC dimensions

UHC Dimension	Primary Cataract Indicator	Secondary Cataract Indicator
**Access (Coverage)** - *the availability of good health services within reasonable reach and available at the point of need.*	Cataract surgical coverage	• Cataract surgical rate[20]<br>• Number of operating surgeons by country<br>• Number and distribution of operating centres by country
**Quality** – *for the purposes of this review, we have limited to the WHO quality elements of effectiveness and safety*	Effective cataract surgical coverage[9]	• Cataract Surgical Outcome (CSO)[12]<br>• Complication Rates per surgeon/institution
**Financial Protection** - *direct payments made to obtain health services do not expose people to financial hardship and do not threaten living standards.*	Rate of Catastrophic Spending on cataract surgery (25% of total household expenditure per WHO)<br>Rate of Impoverishing Spending on cataract surgery (as defined by income PPP/day/capita below country poverty line AND/OR international poverty line)	Cost of Cataract Surgery (to patient / household)[21 22]
**Equity** – *services are available for all who need them*	Disaggregation of any of the primary or secondary indicators by sex/gender[14 16 23]	Disaggregation of any of the primary or secondary indicators by any other PROGRESS factor[24]:<br>**P**lace of residence (e.g. urban/rural, sub-national unit)<br>**R**ace / ethnicity / culture / language<br>**O**ccupation<br>[**G**ender/sex]<br>**Re**ligion<br>**E**ducation<br>**S**ocioeconomic status[13]<br>**S**ocial capital (e.g. marital status)[25]

Search Strategy

We will search Embase, MEDLINE and Global Health databases using search strategies developed by an Information Specialist from Cochrane Eyes and Vision (IG) (MEDLINE Search Strategy included in Annex 1). We will provide a list of included studies and reports to field experts and request they identify additional sources of both published and unpublished reports for consideration in the review.

For peer review only - http://bmjopen.bmj.com/site/about/guidelines.xhtml

We will use the RAAB repository (http://raabdata.info/) to identify all reports and data from sub-national and national RAAB studies taken from January 1, 2000 onwards.

To identify government and non-government reports in the grey literature, we will use a checklist adapted from the Canadian Agency for Drugs and Technologies in Health (CADTH) Grey Matters checklist to undertake a search of relevant websites.[26]

The following grey literature databases and repositories will be searched:

- OpenGrey (http://www.opengrey.eu/)
- Global Burden of Disease (http://www.healthdata.org/gbd)
- Global Health Data Exchange (http://ghdx.healthdata.org/)
- WHO (https://www.who.int/library/en/)
- IAPB (https://www.iapb.org/global-vision-database-maps/)
- National Ministry of Health websites.

Selection of Sources of Evidence

All titles and abstracts will be screened by at least two investigators independently using Covidence systematic review software (Veritas Health Innovation, Melbourne, Australia; available at www.covidence.org). Assessment of eligibility for inclusion will be carried out by two investigators independently with a third investigator reviewing discrepancies. Reference lists of all included articles will be examined to identify further potentially relevant reports. The study selection process will be summarised in a PRISMA flow diagram.

Data Charting and Extraction

The data charting form will be developed in Covidence and piloted by investigators prior to use. As data sources are expected to be heterogenous and broad in nature, data charting will be an iterative process throughout the review. Information that is absent or unclear will be addressed by contacting study authors with up to three attempts by email.

Data Items

Source Characteristics

- Published Data Characteristics - Author(s), Year of Publication, Journal, Language,
- Grey Literature Characteristics - Author (Organisation e.g. WHO, Ministry of Health), Year of Publication, Source Website (e.g. government/non-government organisation), Language, Type of Literature (Report, Thesis, Technical Report, Statistics, other)

For peer review only - http://bmjopen.bmj.com/site/about/guidelines.xhtml

Study characteristics - Type of Study, Countries / regions investigated, Level of analysis (sub-national, national, regional, global), Sample details (frame, size), Year of data collection, Outcome(s) reported (as outlined in Table 1), UHC dimension(s) investigated (Access, Equity, Quality, Financial Protection).

Synthesis of Results

Following data charting, we will undertake narrative synthesis. Where possible maps will be used to summarise the available global, regional, national and sub-national distribution and proportion of studies reporting each UHC dimension for cataract surgery. Tables will be constructed to demonstrate distribution of studies by region and (if appropriate) country. Where enough data are identified, further quantitative analyses of primary or secondary outcomes may be undertaken as a subsequent analysis.

Patient and Public Involvement

This protocol was developed with input from the Commissioners of the *Lancet Global Health* Commission on Global Eye Health,[27] which includes people with lived experience of vision impairment (and cataract surgery), policy makers, academics, clinicians, government eye health programme leaders and advocacy specialists.

Ethics and Dissemination

As this scoping review will only consider publicly available literature and reports, no ethics approval is required. Findings will be published in an open-access peer-reviewed journal, and a summary will be developed for website access and stakeholder meetings. A summary of the findings will also be included in the ongoing *Lancet Global Health* Commission on Global Eye Health.[27]

For peer review only

REFERENCES

1. World Health Organization. World Report on Vision. Geneva: WHO, 2019.
2. World Health Organization. What is universal coverage? [Accessed 1 March 2020]. Available from: http://www.who.int/health_financing/universal_coverage_definition/en/.
3. World Health Organization. Universal coverage - three dimensions [Accessed 1 March 2020]. Available from: https://www.who.int/health_financing/strategy/dimensions/en/.
4. World Health Organization and The World Bank. Tracking universal health coverage: 2017 global monitoring report. Geneva: WHO, 2017.
5. Hogan DR, Stevens GA, Hosseinpoor AR, et al. Monitoring universal health coverage within the Sustainable Development Goals: development and baseline data for an index of essential health services. *The Lancet Global Health* 2018;6(2):e152-e68.
6. World Health Organization. 13th General Programme of Work (GPW13) WHO Impact Framework [Accessed 1 March 2020]. Available from: https://www.who.int/about/what-we-do/GPW13_WHO_Impact_Framework_Indicator_Metadata.pdf.
7. Flaxman S, Bourne R, Resnikoff S, et al. Global Causes of Distance Vision Loss: 1990-2015 and projections to 2020. *Lancet Global Health* 2017;5(12):e1221-e34.
8. Foster A. Cataract and “Vision 2020—the right to sight” initiative. *British Journal of Ophthalmology* 2001;85(6):635-37.
9. Ramke J, Gilbert C, Lee ACL, et al. Effective cataract surgical coverage: an indicator for measuring quality-of-care in the context of Universal Health Coverage *PLoS One* 2017;12(3):e0172342.
10. Ng M, Fullman N, Dieleman JL, et al. Effective coverage: a metric for monitoring universal health coverage. *PLoS Medicine* 2014;11(9):e1001730.
11. World Health Organization. World health statistics 2018: monitoring health for the SDGs, Sustainable Development Goals. Geneva: WHO, 2018.
12. Vijaya L, George R, Rashima A, et al. Outcomes of cataract surgery in a rural and urban south Indian population. *Indian journal of ophthalmology* 2010;58(3):223.
13. Yan W, Wang W, van Wijngaarden P, et al. Longitudinal changes in global cataract surgery rate inequality and associations with socioeconomic indices. *Clinical & experimental ophthalmology* 2019;47(4):453-60.
14. Prasad M, Malhotra S, Kalaivani M, et al. Gender differences in blindness, cataract blindness and cataract surgical coverage in India: a systematic review and meta-analysis. *British Journal of Ophthalmology* 2020;104(2):220-24.
15. Zhao J, Xu X, Ellwein LB, et al. Cataract Surgical Coverage And Visual Acuity Outcomes In Rural China In 2014 And Comparisons With The 2006 China Nine-Province Survey. *American journal of ophthalmology* 2018;193:62-70.
16. Lewallen S, Mousa A, Bassett K, et al. Cataract surgical coverage remains lower in women. *British Journal of Ophthalmology* 2009;93(3):295-98.
17. Munn Z, Peters MD, Stern C, et al. Systematic review or scoping review? Guidance for authors when choosing between a systematic or scoping review approach. *BMC medical research methodology* 2018;18(1):143.
18. Tricco AC, Lillie E, Zarin W, et al. PRISMA extension for scoping reviews (PRISMA-ScR): checklist and explanation. *Annals of internal medicine* 2018;169(7):467-73.
19. World Health Organization. Delivering quality health services: a global imperative for universal health coverage. Geneva: WHO, 2018.
20. Wang W, Yan W, Müller A, et al. A global view on output and outcomes of cataract surgery with national indices of socioeconomic development. *Investigative ophthalmology & visual science* 2017;58(9):3669-76.
21. Ibrahim N, Pozo-Martin F, Gilbert C. Direct non-medical costs double the total direct costs to patients undergoing cataract surgery in Zamfara state, Northern Nigeria: a case series. *BMC Health Services Research* 2015;15(1):163.
22. Fang J, Wang X, Lin Z, et al. Variation of cataract surgery costs in four different graded providers of China. *BMC Public Health* 2010;10(1):543.

For peer review only - http://bmjopen.bmj.com/site/about/guidelines.xhtml

23. Carter MJ, Limburg H, Lansingh VC, et al. Do gender inequities exist in cataract surgical coverage? Meta-analysis in Latin America. *Clinical & Experimental Ophthalmology* 2012;40(5):458-66.
24. O'Neill J, Tabish H, Welch V, et al. Applying an equity lens to interventions: using PROGRESS ensures consideration of socially stratifying factors to illuminate inequities in health. *Journal of Clinical Epidemiology* 2014;67(1):56-64.
25. Ramke J, Kyari F, Mwangi N, et al. Cataract Services are Leaving Widows Behind: Examples from National Cross-Sectional Surveys in Nigeria and Sri Lanka. *International Journal of Environmental Research and Public Health* 2019;16(20):3854.
26. Canadian Agency for Drugs and Technologies in Health. Grey matters: a practical tool for searching health-related grey literature Ottawa2018 [https://www.cadth.ca/resources/finding-evidence.]. Available from: https://www.who.int/about/what-we-do/GPW13_WHO_Impact_Framework_Indicator_Metadata.pdf.
27. Burton MJ, Faal HB, Ramke J, et al. Announcing The Lancet Global Health Commission on Global Eye Health. *The Lancet Global Health* 2019;7:e1612-13.

For peer review only

For peer review only - http://bmjopen.bmj.com/site/about/guidelines.xhtml

Authors' Contributions: JR and MJB conceived the idea for the review. CNL, JR and MJB drafted and revised the protocol with suggestions from IM, JHZ, AA, NM, HB, IG, MY, MH and JCS. IG constructed the search.

Corresponding Author: Jacqueline Ramke (Jacqueline.Ramke@lshtm.ac.uk)

Funding Statement: MJB is supported by the Wellcome Trust (207472/Z/17/Z). JR is a Commonwealth Rutherford Fellow, funded by the UK government through the Commonwealth Scholarship Commission in the UK. The Lancet Global Health Commission on Global Eye Health is supported by The Queen Elizabeth Diamond Jubilee Trust, Moorfields Eye Charity [grant number GR001061], NIHR Moorfields Biomedical Research Centre, Wellcome Trust, Sightsavers, The Fred Hollows Foundation, The SEVA Foundation, British Council for the Prevention of Blindness and Christian Blind Mission.

Competing Interests: None declared

Number of Tables: 1

Number of Figures: 1

Data Sharing Statement: Data generated from this review will be available upon reasonable request from Jacqueline.Ramke@lshtm.ac.uk

Keywords: cataract services, Universal Health Coverage, access, quality, financial protection, equity

Word Count: 1678

For peer review only - http://bmjopen.bmj.com/site/about/guidelines.xhtml

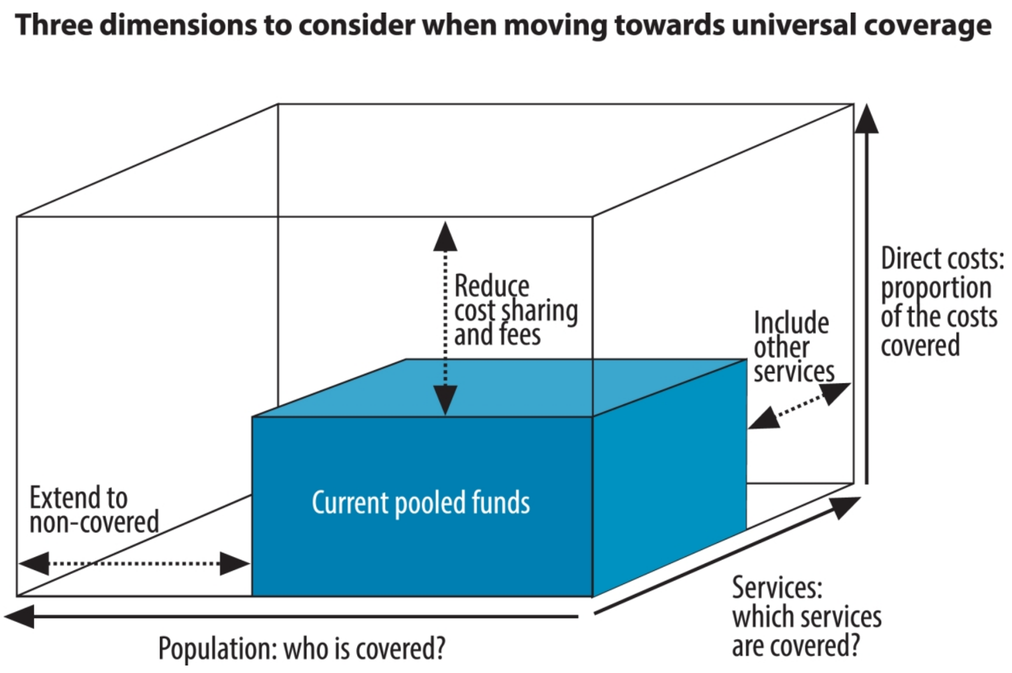

The three dimensions to consider when moving towards universal health coverage

168x110mm (300 x 300 DPI)

Annex 1: Search Results

MEDLINE

1. (cataract$ adj2 surg$ adj2 rate$).tw.
2. (rate$ adj2 cataract$ adj2 surg$).tw.
3. (cataract$ adj2 surg$ adj2 coverage$).tw.
4. (cataract$ adj2 surg$ adj2 outcome$).tw.
5. (incidence adj4 cataract$ adj2 surg$).tw.
6. (rapid adj2 assessment adj3 cataract$).tw.
7. Rapid Assessment of Avoidable Blindness.tw.
8. RAAB.tw.
9. Cataract Extraction/ec [Economics]
10. or/1-9
11. (universal adj2 health adj2 coverag$).tw.
12. cataract$.tw.
13. 11 and 12
14. 10 or 13
15. (cataract$ adj10 (district$ or region$ or province$ or state or states or territor$ or sub-national or national or nation$)).tw.
16. (cataract$ adj10 (country or countries or worldwide or global$ or Asia or China or India or Africa or Europe$)).tw.
17. 15 or 16
18. "Quality of Health Care"/
19. Quality Improvement/
20. Delivery of Health Care/
21. National Health Programs/
22. State Medicine/
23. Regional Health Planning/
24. Health Planning/
25. Health Plan Implementation/
26. Health Planning Guidelines/
27. Health Care Reform/
28. Health Resources/
29. Health Priorities/
30. Health Services Research/
31. "health services needs and demand"/
32. Needs Assessment/
33. State Health Plans/
34. Regional Health Planning/
35. Community Health Planning/
36. Hospital Planning/
37. Regional Medical Programs/
38. Health Maintenance Organizations/
39. Comprehensive Health Care/
40. Health Facility Planning/
41. Health Facility Administration/
42. Hospital Administration/
43. exp Hospitals, public/
44. exp Hospitals, private/
45. health system$.tw.
46. Models, Organizational/
47. Decision Making, Organizational/
48. Resource Allocation/
49. Efficiency, Organizational/
50. Organizational Innovation/
51. Delivery of Health Care, Integrated/
52. Interdisciplinary Communication/
53. Public Health/
54. Health Promotion/
55. Policy Making/
56. Program Development/
57. Program Evaluation/
58. Quality Control/
59. Quality Assurance, Health Care/
60. Benchmarking/
61. Capacity Building/
62. Health Services Accessibility/
63. Health Policy/
64. Surgical Procedures, Operative/
65. exp Surgical Equipment/
66. Health Care Rationing/
67. Medically Underserved Area/
68. Healthcare Disparities/
69. Health Status Disparities/
70. exp Attitude to Health/
71. "Patient Acceptance of Health Care"/
72. Health Education/
73. Public Opinion/
74. Health Behavior/
75. Social Behavior/
76. Superstitions/
77. exp Communication/
78. exp Culture/

For peer review only

79. Sex Factors/
80. (cataract$ adj3 (woman or women or female or gender or sex or sexual or equit$ or inequit$)).tw.
81. Women's Rights/
82. Prejudice/
83. Vulnerable Populations/
84. Social Responsibility/
85. Social Welfare/
86. Urban Health Services/
87. Rural Health Services/
88. Rural Population/
89. Patient Escort Service/
90. Health Manpower/
91. Health Personnel/
92. Health Workforce/
93. Workforce/
94. human resources for eye health.tw.
95. HReH.tw.
96. Medical Staff, Hospital/
97. Nursing Staff, Hospital/
98. Personnel, Hospital/
99. Professional Competence/
100. Clinical Competence/
101. Medical Errors/
102. Clinical Governance/
103. Government Regulation/
104. Public Policy/
105. Public Health Practice/
106. Public Health Administration/
107. Health Plan Implementation/
108. Public-Private Sector Partnerships/
109. Delivery of Health Care, Integrated/
110. service delivery.tw.
111. decision making.tw.
112. (consensus adj3 (process$ or discuss)).tw.
113. stakeholder$.tw.
114. Quality Control/
115. Total Quality Management/
116. Quality Indicators, Health Care/
117. Quality Assurance, Health Care/
118. quality assurance.tw.
119. (quality adj2 improv$).tw.
120. total quality.tw.
121. continuous quality.tw.
122. quality management.tw.
123. (organisation$ adj3 cultur$).tw.
124. Disease Management/
125. Program Evaluation/
126. ((provider$ or program$) adj3 (monitor$ or evaluate$ or modif$ or practice)).tw.
127. (implement$ adj3 (improve$ or change$ or effort$ or issue$ or impede$ or glossary or tool$ or innovation$ or outcome$ or driv$ or examin$ or reexamin$ or scale$ or strateg$ or advis$ or expert$)).tw.
128. (needs adj3 assess$).tw.
129. ((education$ or learn$) adj5 (continu$ or material$ or meeting or collaborat$)).tw.
130. exp Medical audit/
131. (audit or feedback or compliance or adherence or training or innovation).ti.
132. (guideline$ adj3 (clinical or practice or implement$ or promot$)).tw.
133. exp Health Services Accessibility/
134. (outreach adj2 (service$ or visit$)).tw.
135. (intervention$ adj3 (no or usual or routine or target$ or tailor$ or mediat$)).tw.
136. usual care.tw.
137. exp Reminder Systems/
138. remind$.tw.
139. (improve$ adj3 (attend$ or visit$ or intervention$ or adhere$)).tw.
140. (increas$ adj3 (attend$ or visit$ or intervention$ or adhere$)).tw.
141. (appointment$ adj3 (miss$ or fail$ or remind$ or follow up)).tw.
142. Telephone/
143. telephone.tw.
144. Cell Phones/
145. Mobile Applications/
146. Remote Consultation/
147. (m-health or e-health or g-health or u-health).tw.
148. (phone$ adj1 (smart or cell)).tw.
149. (smartphone$ or cellphone$).tw.
150. (hand adj1 held device$).tw.
151. (mobile adj2 (health or healthcare or phone$ or device$ or monitor$ or comput$ or app or apps or application)).tw.
152. Primary Health Care/

For peer review only

153. General Practitioners/ or Physicians, Family/ or Physicians, Primary Care/
154. Primary Prevention/
155. Preventive Health Services/
156. Community Health Services/
157. Community Health Nursing/
158. Health Services, Indigenous/
159. Rural Health Services/
160. Mobile Health Units/
161. (Ophthalmologist$ or Optometrist$ or Optician$ or Orthopist$ or Refractionists).tw.
162. ((Ophthalmic or eye) adj3 (surgeon$ or nurse$ or technician$ or officer$ or assistant$ or staff$)).tw.
163. Physician's Practice Patterns/
164. Professional Practice/
165. (professional adj3 (practice or develop$ or educat)).tw.
166. Education, Medical, Continuing/
167. exp nurses/
168. Specialties, Nursing/
169. Nurse's Role/
170. Education, Nursing, Continuing/
171. (nurse or nurses).tw.
172. ((role or roles) adj3 expan$).tw.
173. (task$ adj3 shift$).tw.
174. exp Medical Records Systems, Computerized/
175. Management Information Systems/
176. Database Management Systems/
177. Computer Systems/
178. Point-of-Care Systems/
179. Hospital Information Systems/
180. ((health or healthcare) adj4 (record or management system$)).tw.
181. (decision adj5 support).ti.
182. Economics/
183. "costs and cost analysis"/
184. Cost allocation/
185. Cost-benefit analysis/
186. Cost control/
187. Cost savings/
188. Cost of illness/
189. Cost sharing/
190. "deductibles and coinsurance"/
191. Medical savings accounts/
192. Health care costs/
193. Direct service costs/
194. Drug costs/
195. Employer health costs/
196. Hospital costs/
197. Health expenditures/
198. Capital expenditures/
199. Value of life/
200. exp economics, hospital/
201. exp economics, medical/
202. Economics, nursing/
203. Economics, pharmaceutical/
204. exp "fees and charges"/
205. exp budgets/
206. (low adj cost).mp.
207. (high adj cost).mp.
208. (health?care adj cost$).mp.
209. (fiscal or funding or financial or finance).tw.
210. (cost adj estimate$).mp.
211. (cost adj variable).mp.
212. (unit adj cost$).mp.
213. (economic$ or pharmacoeconomic$ or price$ or pricing).tw.
214. Uncompensated Care/
215. Reimbursement Mechanisms/
216. Reimbursement, Incentive/
217. (insurance adj3 (health$ or scheme$)).tw.
218. (financial or economic or pay or payment or copayment or paid or fee or fees or monetary or money or cash or incentiv$ or disincentiv$).tw.
219. ((pay or paying or paid or cost$ or free or wait$ or qualit$) adj3 surg$).tw.
220. (will$ adj3 pay$).tw.
221. (waiting adj2 time).tw.
222. ((surgery or surgical or surgeon$ or ophthalmologist$) adj2 (experience$ or supervis$ or rate or rates or output or volume or uptake number$ or coverage or annual$)).tw.
223. Gross Domestic Product/
224. Medicare/
225. human development index.tw.
226. gross domestic product.tw.
227. (HDI or GDP).tw.
228. Cataract Extraction/sn [Statistics & Numerical Data]
229. (global adj2 burden adj2 cataract$).tw.

For peer review only

230. (cataract$ adj3 (cost$ or income$ or price$ or reimburse$)).tw.
231. exp Patient Acceptance of health Care/
232. exp Attitude to Health/
233. exp Health Behavior/
234. Health Education/
235. exp Patient Education as Topic/
236. exp Health Promotion/
237. Socioeconomic Factors/
238. exp Poverty/
239. Social Class/
240. Educational Status/
241. ((school or education$) adj3 (status or level$ or attain$ or achieve$)).tw.
242. Employment/
243. Healthcare Disparities/
244. Health Status Disparities/
245. exp Medically Underserved Area/
246. Rural Population/
247. Urban Population/
248. exp Ethnic Groups/
249. Minority Groups/
250. Vulnerable Populations/
251. ((health$ or social$ or racial$ or ethnic$) adj5 (inequalit$ or inequit$ or disparit$ or equit$ or disadvantage$ or depriv$)).tw.
252. (disadvant$ or marginali$ or underserved or under served or impoverish$ or minorit$ or racial$ or ethnic$).tw.
253. or/18-252
254. 17 and 253
255. 14 or 254
256. (femtosecond or phaco$ or keratometry or vitrectomy or endophthalmitis).ti.
257. (glaucoma$ or intraocular or IOL or keratoplast$ or refractive or retinopathy or tear or uveitis).ti.
258. or/256-257
259. 255 not 258
260. limit 259 to yr="2010 -Current"

For peer review only

Annex 2: PRISMA SCR summary

SECTION	ITEM	PRISMA-ScR CHECKLIST ITEM	REPORTED ON PAGE #
**TITLE**			
Title	1	Identify the report as a scoping review.	1
**ABSTRACT**			
Structured summary	2	Provide a structured summary that includes (as applicable): background, objectives, eligibility criteria, sources of evidence, charting methods, results, and conclusions that relate to the review questions and objectives.	2
**INTRODUCTION**			
Rationale	3	Describe the rationale for the review in the context of what is already known. Explain why the review questions/objectives lend themselves to a scoping review approach.	4-6
Objectives	4	Provide an explicit statement of the questions and objectives being addressed with reference to their key elements (e.g., population or participants, concepts, and context) or other relevant key elements used to conceptualize the review questions and/or objectives.	6
**METHODS**			
Protocol and registration	5	Indicate whether a review protocol exists; state if and where it can be accessed (e.g., a Web address); and if available, provide registration information, including the registration number.	6
Eligibility criteria	6	Specify characteristics of the sources of evidence used as eligibility criteria (e.g., years considered, language, and publication status), and provide a rationale.	6-7
Information sources	7	Describe all information sources in the search (e.g., databases with dates of coverage and contact with authors to identify additional sources), as well as the date the most recent search was executed.	8
Search	8	Present the full electronic search strategy for at least 1 database, including any limits used, such that it could be repeated.	8
Selection of sources of evidence	9	State the process for selecting sources of evidence (i.e., screening and eligibility) included in the scoping review.	8
Data charting process	10	Describe the methods of charting data from the included sources of evidence (e.g., calibrated forms or forms that have been tested by the team before their use, and whether data charting was done independently or in duplicate) and any processes for obtaining and confirming data from investigators.	8-9
Data items	11	List and define all variables for which data were sought and any assumptions and simplifications made.	9
Critical appraisal of individual sources of evidence	12	If done, provide a rationale for conducting a critical appraisal of included sources of evidence; describe the methods used and how this information was used in any data synthesis (if appropriate).	na
Synthesis of results	13	Describe the methods of handling and summarizing the data that were charted.	9
**FUNDING**			
Funding	22	Describe sources of funding for the included sources of evidence, as well as sources of funding for the scoping review. Describe the role of the funders of the scoping review.	12

From: Tricco AC, et al.[18]

For peer review only

BMJ Open

Are we advancing Universal Health Coverage through cataract services? Protocol for a scoping review

Journal:	*BMJ Open*
Manuscript ID	bmjopen-2020-039458.R1
Article Type:	Protocol
Date Submitted by the Author:	27-May-2020
Complete List of Authors:	Lee, Chan; London School of Hygiene and Tropical Medicine, International Centre for Eye Health<br>Ramke, Jacqueline; LSHTM,<br>McCormick, Ian; London School of Hygiene and Tropical Medicine, Department of Clinical Research<br>Zhang, Justine; London School of Hygiene and Tropical Medicine Faculty of Infectious and Tropical Diseases, International Centre for Eye Health, Clinical Research Department<br>Aghaji, Ada; London Sch Hyg ,<br>Mwangi, Nyawira; London School of Hygiene and Tropical Medicine, ; Kenya Medical Training College,<br>Burn, Helen; Brighton and Sussex University Hospitals NHS Trust<br>Gordon, Iris<br>Yusufu, Mayinuer; Beijing Tongren Hospital; Beijing Institute of Ophthalmology<br>He, Mingguang; Sun Yat-sen University, State Key Laboratory of Ophthalmology, Zhongshan Ophthalmic Center; University of Melbourne, Centre for Eye Research Australia, Royal Victorian Eye and Ear Hospital<br>Silva, Juan Carlos; Pan American Health Organization, Life Course ; PAHO<br>Burton, Matthew J; London School of Hygiene and Tropical Medicine
**Primary Subject Heading**:	Ophthalmology
Secondary Subject Heading:	Epidemiology, Global health, Surgery, Health services research, Health informatics
Keywords:	Cataract and refractive surgery < OPHTHALMOLOGY, EPIDEMIOLOGY, PUBLIC HEALTH, Adult surgery < SURGERY, Ophthalmology < SURGERY

SCHOLARONE™
Manuscripts

I, the Submitting Author has the right to grant and does grant on behalf of all authors of the Work (as defined in the below author licence), an exclusive licence and/or a non-exclusive licence for contributions from authors who are: i) UK Crown employees; ii) where BMJ has agreed a CC-BY licence shall apply, and/or iii) in accordance with the terms applicable for US Federal Government officers or employees acting as part of their official duties; on a worldwide, perpetual, irrevocable, royalty-free basis to BMJ Publishing Group Ltd ("BMJ") its licensees and where the relevant Journal is co-owned by BMJ to the co-owners of the Journal, to publish the Work in this journal and any other BMJ products and to exploit all rights, as set out in our licence.

The Submitting Author accepts and understands that any supply made under these terms is made by BMJ to the Submitting Author unless you are acting as an employee on behalf of your employer or a postgraduate student of an affiliated institution which is paying any applicable article publishing charge ("APC") for Open Access articles. Where the Submitting Author wishes to make the Work available on an Open Access basis (and intends to pay the relevant APC), the terms of reuse of such Open Access shall be governed by a Creative Commons licence – details of these licences and which Creative Commons licence will apply to this Work are set out in our licence referred to above.

Other than as permitted in any relevant BMJ Author's Self Archiving Policies, I confirm this Work has not been accepted for publication elsewhere, is not being considered for publication elsewhere and does not duplicate material already published. I confirm all authors consent to publication of this Work and authorise the granting of this licence.

For peer review only

Are we advancing Universal Health Coverage through cataract services? Protocol for a scoping review

Authors:

Chan Ning Lee[1,2], Jacqueline Ramke[1,3], Ian McCormick[1], Justine H. Zhang[1,4], Ada Aghaji [1,5], Nyawira Mwangi[1,6], Helen Burn[1,7], Iris Gordon[1], Mayinuer Yusufu[8], Mingguang He[9], Juan Carlos Silva[10], Matthew J Burton[1,11]

Affiliations

1. International Centre for Eye Health, London School of Hygiene and Tropical Medicine, London, United Kingdom
2. St Paul's Eye Unit, Royal Liverpool University Hospital, Liverpool, United Kingdom
3. School of Optometry and Vision Science, University of Auckland, Auckland, New Zealand
4. Manchester Royal Eye Hospital, Manchester, United Kingdom
5. Department of Opthalmology, College of Medicine, University of Nigeria, Enugu, Nigeria
6. Department of Clinical Medicine, Kenya Medical Training College, Nairobi, Kenya
7. Beijing Institute of Ophthalmology, Beijing Tongren Eye Center, Beijing Tongren Hospital, Capital Medical University, Beijing, China
8. Department of Ophthalmology, Stoke Mandeville Hospital, Aylesbury, United Kingdom
9. Centre for Eye Research Australia, University of Melbourne, Royal Victorian Eye and Ear Hospital, East Melbourne, Australia
10. Division of Blindness Prevention, Pan American Health Organization, Bogota, Colombia
11. Moorfields Eye Hospital, London, United Kingdom

For peer review only - http://bmjopen.bmj.com/site/about/guidelines.xhtml

ABSTRACT

Introduction

Universal Health Coverage (UHC) includes the dimensions of equity in access, quality services that improve health, and protection against financial hardship. Cataract continues to be the leading cause of blindness globally, despite cataract surgery being an efficacious intervention. The aim of this scoping review is to map the nature, extent and global distribution of data on cataract services for UHC in terms of equity, access, quality and financial protection.

Methods and analysis

The search will be constructed by an Information Specialist and undertaken in MEDLINE, Embase and Global Health databases. We will include all published non-interventional primary research studies and systematic reviews that report a quantitative assessment of access, equity, quality or financial protection of cataract surgical services for adults at the sub-national, national, regional, or global level from population-based surveys or routinely collected health service data since 1 January 2000.
Screening and data charting will be undertaken using Covidence systematic review software. Titles and abstracts of identified studies will be screened by two authors independently. Full text articles of potentially relevant studies will be obtained and reviewed independently by two authors against the inclusion criteria. Any discrepancies between the authors will be resolved by discussion, with a third author as necessary. A data charting form will be developed and piloted on three studies by three authors, and amendments made as necessary. Data will be extracted by two reviewers independently and summarised narratively and using maps.

Ethics and dissemination

Ethical approval was not sought as the scoping review will only use published and publicly accessible data. The review will be published in an open access peer-reviewed journal. A summary of the results will be developed for website posting, stakeholder meetings, and inclusion in the ongoing *Lancet Global Health* Commission on Global Eye Health.

For peer review only

For peer review only - http://bmjopen.bmj.com/site/about/guidelines.xhtml

STRENGTHS AND LIMITATIONS

- The broad scope of this review will result in the first synthesis to date of data on the UHC dimensions of cataract surgical services.
- Another strength is that we will include studies from all world regions and high-, low- and middle-income countries with no language restrictions, to give a global picture of cataract services.
- A potential limitation is the paucity of available information on the 'financial protection' dimension.

For peer review only

For peer review only - http://bmjopen.bmj.com/site/about/guidelines.xhtml

INTRODUCTION

Eye health and vision impairment represent a major global health concern. In the recent *World Report on Vision,* the World Health Organization (WHO) outlined how the provision of quality eye care services contributes directly to achieving Universal Health Coverage (UHC).[1] WHO estimated that in 2020 up to 2.2 billion people have some form of vision impairment and that this figure is projected to rise leading to an increased burden on health systems.[1]

Monitoring progress towards UHC

The WHO defines UHC in the following terms:

"Universal health coverage (UHC) means that all people and communities can use the promotive, preventive, curative, rehabilitative and palliative health services they need, of sufficient quality to be effective, while also ensuring that the use of these services does not expose the user to financial hardship".[2]

It has three broad principles: (1) equity in access; (2) quality services that improve health; (3) protection against financial risk. WHO and the World Bank have developed a framework for tracking progress towards UHC.[3] This focuses on two elements:

1. measuring the coverage of essential health services, as a proportion of the population that can access essential quality health services; and
2. measuring financial protection by determining the proportion of the population in whom direct payment made to obtain health services leads to financial hardship and/or a threat to living standards.[3]

A variety of different types of indicators are used. *Effective service coverage indicators* measure the proportion of people in need of services who receive services of sufficient quality to obtain potential health gains; these are preferred if available. *Service coverage indicators* measure the proportion of the population that needs a service that receive it. *Proxy indicators* are sometimes used where service coverage indicators are not available, but provide a correlated indication of the provision of a health service.[3 4]

The WHO and World Bank have selected a panel of 16 "tracer indicators" to monitor progress towards UHC.[2] There is currently no eye health related indicator among this panel though *effective cataract surgical coverage* and *effective refractive error coverage* were proposed in WHO's Thirteenth General Programme of Work 2019 – 2023 Impact Framework.[5] In addition to measuring population level coverage, it is very important to measure equity in service provision, by disaggregating the data and comparing sub-populations such as wealth quintiles, education, sex, age and geographical region.[3]

For peer review only - http://bmjopen.bmj.com/site/about/guidelines.xhtml

Monitoring cataract services for UHC

Cataract is the leading cause of blindness globally and is the second leading cause of vision impairment.[6] The last three decades have seen a marked increase in available data on vision impairment due to cataract, as well as cataract services. These data have enabled calculation of indicators of *access* and *quality* of cataract surgery, including:

- Cataract Surgical Rate (CSR): the number of cataract operations per million population per year.[7]
- Cataract Surgical Coverage (CSC): the number of people in a population who have received cataract surgery as a proportion of those having operable and operated cataract.[6]
- Cataract Surgical Outcome (CSO): the presenting visual acuity of the operated eye.
- Effective cataract surgical coverage (eCSC): the number of people in a population with operated cataract and a visual acuity of 6/18 or better as a proportion of those having operable and operated cataract.[8]

Effective Cataract Surgical Coverage (eCSC), has the characteristics of an *Effective service coverage indicator,* as preferred by the WHO/World Bank, as it combines information on the proportion of the population covered and the outcome of the surgical intervention.[9 10]

Disaggregation of larger datasets has allowed analyses of *equity* in cataract surgery as well, for example in highlighting existing gender disparities in CSC.[11-15] Much less data are available quantifying financial aspects of cataract services. To our knowledge, no existing synthesis of the distribution and quantity of known evidence for the UHC dimensions of cataract surgery has been undertaken. The aim of this scoping review is to map the nature, extent and global distribution of data on cataract surgical services for UHC in terms of equity, access, quality and financial protection.

We chose to undertake a scoping review rather than an alternative evidence synthesis approach because we wished to identify and map the available evidence, which we anticipate will be heterogeneous.[16]

METHODS

Objectives / Scoping review questions

We aim to answer the following two questions in relation to cataract services for UHC:

1. What is the nature, extent and global distribution of data on the coverage and effectiveness of cataract services?
2. What is the nature, extent and global distribution of data on financial protection in relation to cataract services?

For peer review only - http://bmjopen.bmj.com/site/about/guidelines.xhtml

Protocol and registration

The protocol for this scoping review is reported according to the relevant sections of the PRISMA Extension for Scoping Reviews (PRISMA-ScR) guideline (Annex 1).[17] The protocol is registered on the Open Science Framework (https://osf.io/k3mwg/).

Eligibility Criteria

We will include all published prospective and retrospective primary research studies and systematic reviews that report a quantitative assessment of access, equity, quality or financial protection of cataract surgical services for adults at the sub-national, national, regional, or global level (examples outlined in Table 1). We will include population-level observational studies and reports, including those that use routinely collected data (such as in health information systems) and household surveys such as Rapid Assessment of Avoidable Blindness (RAAB) surveys. We will exclude intervention studies and studies within clinical sub-populations as their outcomes can be different to the general population (e.g. people with diabetes, people with age related macular degeneration). We will exclude studies focused exclusively on cataract services for children (aged under 18 years), as these services differ substantially from those for age-related cataract.

To assess *access* we will include studies that report cataract surgical coverage and cataract surgical rate, which are priority indicators for monitoring global eye health.[18] Beyond these, we will include studies that report the number and distribution of human resources and surgical facilities. We acknowledge that there are many quantitative and qualitative elements of health care *quality* as defined by the WHO.[19] However, for the purposes of this review we will focus on only three—effective cataract surgical coverage, vision outcomes of cataract surgery and reported complications. We anticipate the literature reporting *financial protection* associated with cataract surgery will be limited. We will include studies and surveys that report 'catastrophic' and 'impoverishing' spending on cataract surgery according to WHO definitions, as well as other related measures of personal and government expenditure on cataract surgery and service provision (Table 1). We will use the PROGRESS acronym[20] to assess *equity*.

Studies will be limited to those including data collected since 1 January 2000 to provide a contemporary view of cataract services. The search strategy will be undertaken without language restrictions and translation will be arranged when required.

For peer review only - http://bmjopen.bmj.com/site/about/guidelines.xhtml

Table 1: Primary and secondary outcomes included in the review, mapped against UHC dimensions

UHC Dimension	Primary Cataract Indicator	Secondary Cataract Indicator
**Access (Coverage)** - *the availability of good health services within reasonable reach and available at the point of need.*	Cataract surgical coverage	• Cataract surgical rate[21]<br>• Number of operating surgeons by country<br>• Number and distribution of operating centres by country
**Quality** – *limited to the WHO quality elements of effectiveness and safety*	Effective cataract surgical coverage[8]	• Cataract Surgical Outcome (CSO)[11]<br>• Complication Rates per surgeon/institution
**Financial Protection** - *direct payments made to obtain health services do not expose people to financial hardship and do not threaten living standards.*	Rate of Catastrophic Spending on cataract surgery (25% of total household expenditure per WHO)<br>Rate of Impoverishing Spending on cataract surgery (as defined by income PPP/day/capita below country poverty line AND/OR international poverty line)	Cost of Cataract Surgery (to patient / household)[22 23]
**Equity** – *services are available for all who need them*	Disaggregation of any of the primary or secondary indicators by sex/gender[13 15 24]	Disaggregation of any of the primary or secondary indicators by any other PROGRESS factor[20]:<br>**P**lace of residence (e.g. urban/rural, sub-national unit)<br>**R**ace / ethnicity / culture / language<br>**O**ccupation<br>[**G**ender/sex]<br>**R**eligion<br>**E**ducation<br>**S**ocioeconomic status[12]<br>**S**ocial capital (e.g. marital status)[25]

Search Strategy

We will search Embase, MEDLINE and Global Health databases for studies published from 1 January 2000 through to February 2020 using search strategies developed by an Information Specialist from Cochrane Eyes and Vision (IG) (MEDLINE Search Strategy included in Annex 2). We will provide a list of included studies and reports to field experts and request they identify additional sources of both published and unpublished reports for consideration in the review.

We will use the RAAB repository (http://raabdata.info/) to identify all reports and data from sub-national and national RAAB studies taken from January 1, 2000 onwards.

To identify government and non-government reports in the grey literature, we will use a checklist adapted from the Canadian Agency for Drugs and Technologies in Health (CADTH) Grey Matters checklist to undertake a search of relevant websites.[26]

The following grey literature databases and repositories will be searched:

- OpenGrey (http://www.opengrey.eu/)
- Global Burden of Disease (http://www.healthdata.org/gbd)
- Global Health Data Exchange (http://ghdx.healthdata.org/)
- WHO (https://www.who.int/library/en/)

For peer review only - http://bmjopen.bmj.com/site/about/guidelines.xhtml

- IAPB (https://www.iapb.org/global-vision-database-maps/)
- National Ministry of Health websites.

Selection of Sources of Evidence

All titles and abstracts will be screened by at least two investigators independently using Covidence systematic review software (Veritas Health Innovation, Melbourne, Australia; available at www.covidence.org). Assessment of eligibility for inclusion will be carried out by two investigators independently with a third investigator reviewing discrepancies. Reference lists of all included articles will be examined to identify further potentially relevant reports. The study selection process will be summarised in a PRISMA flow diagram.

Data Charting

The data charting form will be developed in Covidence and piloted by investigators prior to use. Data charting will be carried out by two investigators independently. As data sources are expected to be heterogenous and broad in nature, data charting will be an iterative process throughout the review. Information that is absent or unclear will be addressed by contacting study authors with up to three attempts by email.

Data Items

Source Characteristics

- Published Data Characteristics - Author(s), Year of Publication, Journal, Language,
- Grey Literature Characteristics - Author (Organisation e.g. WHO, Ministry of Health), Year of Publication, Source Website (e.g. government/non-government organisation), Language, Type of Literature (Report, Thesis, Technical Report, Statistics, other)

Study characteristics - Type of Study, Countries / regions investigated, Level of analysis (sub-national, national, regional, global), Sample details (frame, size), Year of data collection, Outcome(s) reported (as outlined in Table 1), UHC dimension(s) investigated (Access, Equity, Quality, Financial Protection).

Synthesis of Results

Following data charting, we will undertake narrative synthesis. Where possible maps will be used to summarise the available global, regional, national and sub-national distribution and proportion of studies reporting each UHC dimension for cataract surgery. Tables will be constructed to demonstrate distribution of studies by region and (if appropriate) country. Where enough data are identified,

For peer review only - http://bmjopen.bmj.com/site/about/guidelines.xhtml

further quantitative analyses of primary or secondary outcomes may be undertaken as a subsequent analysis.

Patient and Public Involvement

This protocol was developed with input from the Commissioners of the *Lancet Global Health* Commission on Global Eye Health,[27] which includes people with lived experience of vision impairment (and cataract surgery), policy makers, academics, clinicians, government eye health programme leaders and advocacy specialists.

Ethics and Dissemination

As this scoping review will only consider publicly available literature and reports, no ethics approval is required. Findings will be published in an open-access peer-reviewed journal, and a summary will be developed for website access and stakeholder meetings. A summary of the findings will also be included in the ongoing *Lancet Global Health* Commission on Global Eye Health.[27]

For peer review only

For peer review only - http://bmjopen.bmj.com/site/about/guidelines.xhtml

REFERENCES

1. World Health Organization. World Report on Vision. Geneva: WHO, 2019.
2. World Health Organization. What is universal coverage? [Accessed 1 March 2020]. Available from: http://www.who.int/health_financing/universal_coverage_definition/en/.
3. World Health Organization and The World Bank. Tracking universal health coverage: 2017 global monitoring report. Geneva: WHO, 2017.
4. Hogan DR, Stevens GA, Hosseinpoor AR, et al. Monitoring universal health coverage within the Sustainable Development Goals: development and baseline data for an index of essential health services. *The Lancet Global Health* 2018;6(2):e152-e68.
5. World Health Organization. 13th General Programme of Work (GPW13) WHO Impact Framework [Accessed 1 March 2020]. Available from: https://www.who.int/about/what-we-do/GPW13_WHO_Impact_Framework_Indicator_Metadata.pdf.
6. Flaxman S, Bourne R, Resnikoff S, et al. Global Causes of Distance Vision Loss: 1990-2015 and projections to 2020. *Lancet Global Health* 2017;5(12):e1221-e34.
7. Foster A. Cataract and “Vision 2020—the right to sight” initiative. *British Journal of Ophthalmology* 2001;85(6):635-37.
8. Ramke J, Gilbert C, Lee ACL, et al. Effective cataract surgical coverage: an indicator for measuring quality-of-care in the context of Universal Health Coverage *PLoS One* 2017;12(3):e0172342.
9. Ng M, Fullman N, Dieleman JL, et al. Effective coverage: a metric for monitoring universal health coverage. *PLoS Medicine* 2014;11(9):e1001730.
10. World Health Organization. World health statistics 2018: monitoring health for the SDGs, Sustainable Development Goals. Geneva: WHO, 2018.
11. Vijaya L, George R, Rashima A, et al. Outcomes of cataract surgery in a rural and urban south Indian population. *Indian Journal of Ophthalmology* 2010;58(3):223.
12. Yan W, Wang W, van Wijngaarden P, et al. Longitudinal changes in global cataract surgery rate inequality and associations with socioeconomic indices. *Clinical & Experimental Ophthalmology* 2019;47(4):453-60.
13. Prasad M, Malhotra S, Kalaivani M, et al. Gender differences in blindness, cataract blindness and cataract surgical coverage in India: a systematic review and meta-analysis. *British Journal of Ophthalmology* 2020;104(2):220-24.
14. Zhao J, Xu X, Ellwein LB, et al. Cataract Surgical Coverage And Visual Acuity Outcomes In Rural China In 2014 And Comparisons With The 2006 China Nine-Province Survey. *American Journal of Ophthalmology* 2018;193:62-70.
15. Lewallen S, Mousa A, Bassett K, et al. Cataract surgical coverage remains lower in women. *British Journal of Ophthalmology* 2009;93(3):295-98.
16. Munn Z, Peters MD, Stern C, et al. Systematic review or scoping review? Guidance for authors when choosing between a systematic or scoping review approach. *BMC Medical Research Methodology* 2018;18(1):143.
17. Tricco AC, Lillie E, Zarin W, et al. PRISMA extension for scoping reviews (PRISMA-ScR): checklist and explanation. *Annals of Internal Medicine* 2018;169(7):467-73.
18. World Health Organization. Universal Eye Health: A global action plan 2014-2019. Geneva: WHO, 2013.
19. World Health Organization. Delivering quality health services: a global imperative for universal health coverage. Geneva: WHO, 2018.
20. O'Neill J, Tabish H, Welch V, et al. Applying an equity lens to interventions: using PROGRESS ensures consideration of socially stratifying factors to illuminate inequities in health. *Journal of Clinical Epidemiology* 2014;67(1):56-64.
21. Wang W, Yan W, Müller A, et al. A global view on output and outcomes of cataract surgery with national indices of socioeconomic development. *Investigative Ophthalmology & Visual Science* 2017;58(9):3669-76.
22. Ibrahim N, Pozo-Martin F, Gilbert C. Direct non-medical costs double the total direct costs to patients undergoing cataract surgery in Zamfara state, Northern Nigeria: a case series. *BMC Health Services Research* 2015;15(1):163.

For peer review only - http://bmjopen.bmj.com/site/about/guidelines.xhtml

23. Fang J, Wang X, Lin Z, et al. Variation of cataract surgery costs in four different graded providers of China. *BMC Public Health* 2010;10(1):543.
24. Carter MJ, Limburg H, Lansingh VC, et al. Do gender inequities exist in cataract surgical coverage? Meta-analysis in Latin America. *Clinical & Experimental Ophthalmology* 2012;40(5):458-66.
25. Ramke J, Kyari F, Mwangi N, et al. Cataract Services are Leaving Widows Behind: Examples from National Cross-Sectional Surveys in Nigeria and Sri Lanka. *International Journal of Environmental Research and Public Health* 2019;16(20):3854.
26. Canadian Agency for Drugs and Technologies in Health. Grey matters: a practical tool for searching health-related grey literature Ottawa2018 [https://www.cadth.ca/resources/finding-evidence.]. Available from: https://www.who.int/about/what-we-do/GPW13_WHO_Impact_Framework_Indicator_Metadata.pdf.
27. Burton MJ, Faal HB, Ramke J, et al. Announcing The Lancet Global Health Commission on Global Eye Health. *The Lancet Global Health* 2019;7:e1612-13.

For peer review only

For peer review only - http://bmjopen.bmj.com/site/about/guidelines.xhtml

Authors' Contributions: JR and MJB conceived the idea for the review. CNL, JR and MJB drafted and revised the protocol with suggestions from IM, JHZ, AA, NM, HB, IG, MY, MH and JCS. IG constructed the search.

Corresponding Author: Jacqueline Ramke (Jacqueline.Ramke@lshtm.ac.uk)

Funding Statement: MJB is supported by the Wellcome Trust (207472/Z/17/Z). JR is a Commonwealth Rutherford Fellow, funded by the UK government through the Commonwealth Scholarship Commission in the UK. The Lancet Global Health Commission on Global Eye Health is supported by The Queen Elizabeth Diamond Jubilee Trust, Moorfields Eye Charity [grant number GR001061], NIHR Moorfields Biomedical Research Centre, Wellcome Trust, Sightsavers, The Fred Hollows Foundation, The SEVA Foundation, British Council for the Prevention of Blindness and Christian Blind Mission.

Competing Interests: None declared

Number of Tables: 1

Number of Figures: 0

Data Sharing Statement: Data generated from this review will be available upon reasonable request from Jacqueline.Ramke@lshtm.ac.uk

Keywords: cataract services, Universal Health Coverage, access, quality, financial protection, equity

Word Count: 1686

For peer review only

Annex 2: PRISMA SCR summary

SECTION	ITEM	PRISMA-ScR CHECKLIST ITEM	REPORTED ON PAGE #
**TITLE**			
Title	1	Identify the report as a scoping review.	1
**ABSTRACT**			
Structured summary	2	Provide a structured summary that includes (as applicable): background, objectives, eligibility criteria, sources of evidence, charting methods, results, and conclusions that relate to the review questions and objectives.	2
**INTRODUCTION**			
Rationale	3	Describe the rationale for the review in the context of what is already known. Explain why the review questions/objectives lend themselves to a scoping review approach.	4-6
Objectives	4	Provide an explicit statement of the questions and objectives being addressed with reference to their key elements (e.g., population or participants, concepts, and context) or other relevant key elements used to conceptualize the review questions and/or objectives.	6
**METHODS**			
Protocol and registration	5	Indicate whether a review protocol exists; state if and where it can be accessed (e.g., a Web address); and if available, provide registration information, including the registration number.	6
Eligibility criteria	6	Specify characteristics of the sources of evidence used as eligibility criteria (e.g., years considered, language, and publication status), and provide a rationale.	6-7
Information sources	7	Describe all information sources in the search (e.g., databases with dates of coverage and contact with authors to identify additional sources), as well as the date the most recent search was executed.	8
Search	8	Present the full electronic search strategy for at least 1 database, including any limits used, such that it could be repeated.	8
Selection of sources of evidence	9	State the process for selecting sources of evidence (i.e., screening and eligibility) included in the scoping review.	8
Data charting process	10	Describe the methods of charting data from the included sources of evidence (e.g., calibrated forms or forms that have been tested by the team before their use, and whether data charting was done independently or in duplicate) and any processes for obtaining and confirming data from investigators.	8-9
Data items	11	List and define all variables for which data were sought and any assumptions and simplifications made.	9
Critical appraisal of individual sources of evidence	12	If done, provide a rationale for conducting a critical appraisal of included sources of evidence; describe the methods used and how this information was used in any data synthesis (if appropriate).	na
Synthesis of results	13	Describe the methods of handling and summarizing the data that were charted.	9
**FUNDING**			
Funding	22	Describe sources of funding for the included sources of evidence, as well as sources of funding for the scoping review. Describe the role of the funders of the scoping review.	12

From: Tricco AC, et al.[18]

For peer review only

Annex 1: Search Results

MEDLINE

1. (cataract$ adj2 surg$ adj2 rate$).tw.
2. (rate$ adj2 cataract$ adj2 surg$).tw.
3. (cataract$ adj2 surg$ adj2 coverage$).tw.
4. (cataract$ adj2 surg$ adj2 outcome$).tw.
5. (incidence adj4 cataract$ adj2 surg$).tw.
6. (rapid adj2 assessment adj3 cataract$).tw.
7. Rapid Assessment of Avoidable Blindness.tw.
8. RAAB.tw.
9. Cataract Extraction/ec [Economics]
10. or/1-9
11. (universal adj2 health adj2 coverag$).tw.
12. cataract$.tw.
13. 11 and 12
14. 10 or 13
15. (cataract$ adj10 (district$ or region$ or province$ or state or states or territor$ or sub-national or national or nation$)).tw.
16. (cataract$ adj10 (country or countries or worldwide or global$ or Asia or China or India or Africa or Europe$)).tw.
17. 15 or 16
18. "Quality of Health Care"/
19. Quality Improvement/
20. Delivery of Health Care/
21. National Health Programs/
22. State Medicine/
23. Regional Health Planning/
24. Health Planning/
25. Health Plan Implementation/
26. Health Planning Guidelines/
27. Health Care Reform/
28. Health Resources/
29. Health Priorities/
30. Health Services Research/
31. "health services needs and demand"/
32. Needs Assessment/
33. State Health Plans/
34. Regional Health Planning/
35. Community Health Planning/
36. Hospital Planning/
37. Regional Medical Programs/
38. Health Maintenance Organizations/
39. Comprehensive Health Care/
40. Health Facility Planning/
41. Health Facility Administration/
42. Hospital Administration/
43. exp Hospitals, public/
44. exp Hospitals, private/
45. health system$.tw.
46. Models, Organizational/
47. Decision Making, Organizational/
48. Resource Allocation/
49. Efficiency, Organizational/
50. Organizational Innovation/
51. Delivery of Health Care, Integrated/
52. Interdisciplinary Communication/
53. Public Health/
54. Health Promotion/
55. Policy Making/
56. Program Development/
57. Program Evaluation/
58. Quality Control/
59. Quality Assurance, Health Care/
60. Benchmarking/
61. Capacity Building/
62. Health Services Accessibility/
63. Health Policy/
64. Surgical Procedures, Operative/
65. exp Surgical Equipment/
66. Health Care Rationing/
67. Medically Underserved Area/
68. Healthcare Disparities/
69. Health Status Disparities/
70. exp Attitude to Health/
71. "Patient Acceptance of Health Care"/
72. Health Education/
73. Public Opinion/
74. Health Behavior/
75. Social Behavior/
76. Superstitions/
77. exp Communication/
78. exp Culture/

For peer review only

79. Sex Factors/
80. (cataract$ adj3 (woman or women or female or gender or sex or sexual or equit$ or inequit$)).tw.
81. Women's Rights/
82. Prejudice/
83. Vulnerable Populations/
84. Social Responsibility/
85. Social Welfare/
86. Urban Health Services/
87. Rural Health Services/
88. Rural Population/
89. Patient Escort Service/
90. Health Manpower/
91. Health Personnel/
92. Health Workforce/
93. Workforce/
94. human resources for eye health.tw.
95. HReH.tw.
96. Medical Staff, Hospital/
97. Nursing Staff, Hospital/
98. Personnel, Hospital/
99. Professional Competence/
100. Clinical Competence/
101. Medical Errors/
102. Clinical Governance/
103. Government Regulation/
104. Public Policy/
105. Public Health Practice/
106. Public Health Administration/
107. Health Plan Implementation/
108. Public-Private Sector Partnerships/
109. Delivery of Health Care, Integrated/
110. service delivery.tw.
111. decision making.tw.
112. (consensus adj3 (process$ or discuss)).tw.
113. stakeholder$.tw.
114. Quality Control/
115. Total Quality Management/
116. Quality Indicators, Health Care/
117. Quality Assurance, Health Care/
118. quality assurance.tw.
119. (quality adj2 improv$).tw.
120. total quality.tw.
121. continuous quality.tw.
122. quality management.tw.
123. (organisation$ adj3 cultur$).tw.
124. Disease Management/
125. Program Evaluation/
126. ((provider$ or program$) adj3 (monitor$ or evaluate$ or modif$ or practice)).tw.
127. (implement$ adj3 (improve$ or change$ or effort$ or issue$ or impede$ or glossary or tool$ or innovation$ or outcome$ or driv$ or examin$ or reexamin$ or scale$ or strateg$ or advis$ or expert$)).tw.
128. (needs adj3 assess$).tw.
129. ((education$ or learn$) adj5 (continu$ or material$ or meeting or collaborat$)).tw.
130. exp Medical audit/
131. (audit or feedback or compliance or adherence or training or innovation).ti.
132. (guideline$ adj3 (clinical or practice or implement$ or promot$)).tw.
133. exp Health Services Accessibility/
134. (outreach adj2 (service$ or visit$)).tw.
135. (intervention$ adj3 (no or usual or routine or target$ or tailor$ or mediat$)).tw.
136. usual care.tw.
137. exp Reminder Systems/
138. remind$.tw.
139. (improve$ adj3 (attend$ or visit$ or intervention$ or adhere$)).tw.
140. (increas$ adj3 (attend$ or visit$ or intervention$ or adhere$)).tw.
141. (appointment$ adj3 (miss$ or fail$ or remind$ or follow up)).tw.
142. Telephone/
143. telephone.tw.
144. Cell Phones/
145. Mobile Applications/
146. Remote Consultation/
147. (m-health or e-health or g-health or u-health).tw.
148. (phone$ adj1 (smart or cell)).tw.
149. (smartphone$ or cellphone$).tw.
150. (hand adj1 held device$).tw.
151. (mobile adj2 (health or healthcare or phone$ or device$ or monitor$ or comput$ or app or apps or application)).tw.
152. Primary Health Care/

For peer review only

153. General Practitioners/ or Physicians, Family/ or Physicians, Primary Care/
154. Primary Prevention/
155. Preventive Health Services/
156. Community Health Services/
157. Community Health Nursing/
158. Health Services, Indigenous/
159. Rural Health Services/
160. Mobile Health Units/
161. (Ophthalmologist$ or Optometrist$ or Optician$ or Orthopist$ or Refractionists).tw.
162. ((Ophthalmic or eye) adj3 (surgeon$ or nurse$ or technician$ or officer$ or assistant$ or staff$)).tw.
163. Physician's Practice Patterns/
164. Professional Practice/
165. (professional adj3 (practice or develop$ or educat)).tw.
166. Education, Medical, Continuing/
167. exp nurses/
168. Specialties, Nursing/
169. Nurse's Role/
170. Education, Nursing, Continuing/
171. (nurse or nurses).tw.
172. ((role or roles) adj3 expan$).tw.
173. (task$ adj3 shift$).tw.
174. exp Medical Records Systems, Computerized/
175. Management Information Systems/
176. Database Management Systems/
177. Computer Systems/
178. Point-of-Care Systems/
179. Hospital Information Systems/
180. ((health or healthcare) adj4 (record or management system$)).tw.
181. (decision adj5 support).ti.
182. Economics/
183. "costs and cost analysis"/
184. Cost allocation/
185. Cost-benefit analysis/
186. Cost control/
187. Cost savings/
188. Cost of illness/
189. Cost sharing/
190. "deductibles and coinsurance"/
191. Medical savings accounts/
192. Health care costs/
193. Direct service costs/
194. Drug costs/
195. Employer health costs/
196. Hospital costs/
197. Health expenditures/
198. Capital expenditures/
199. Value of life/
200. exp economics, hospital/
201. exp economics, medical/
202. Economics, nursing/
203. Economics, pharmaceutical/
204. exp "fees and charges"/
205. exp budgets/
206. (low adj cost).mp.
207. (high adj cost).mp.
208. (health?care adj cost$).mp.
209. (fiscal or funding or financial or finance).tw.
210. (cost adj estimate$).mp.
211. (cost adj variable).mp.
212. (unit adj cost$).mp.
213. (economic$ or pharmacoeconomic$ or price$ or pricing).tw.
214. Uncompensated Care/
215. Reimbursement Mechanisms/
216. Reimbursement, Incentive/
217. (insurance adj3 (health$ or scheme$)).tw.
218. (financial or economic or pay or payment or copayment or paid or fee or fees or monetary or money or cash or incentiv$ or disincentiv$).tw.
219. ((pay or paying or paid or cost$ or free or wait$ or qualit$) adj3 surg$).tw.
220. (will$ adj3 pay$).tw.
221. (waiting adj2 time).tw.
222. ((surgery or surgical or surgeon$ or ophthalmologist$) adj2 (experience$ or supervis$ or rate or rates or output or volume or uptake number$ or coverage or annual$)).tw.
223. Gross Domestic Product/
224. Medicare/
225. human development index.tw.
226. gross domestic product.tw.
227. (HDI or GDP).tw.
228. Cataract Extraction/sn [Statistics & Numerical Data]
229. (global adj2 burden adj2 cataract$).tw.

For peer review only

230. (cataract$ adj3 (cost$ or income$ or price$ or reimburse$)).tw.
231. exp Patient Acceptance of health Care/
232. exp Attitude to Health/
233. exp Health Behavior/
234. Health Education/
235. exp Patient Education as Topic/
236. exp Health Promotion/
237. Socioeconomic Factors/
238. exp Poverty/
239. Social Class/
240. Educational Status/
241. ((school or education$) adj3 (status or level$ or attain$ or achieve$)).tw.
242. Employment/
243. Healthcare Disparities/
244. Health Status Disparities/
245. exp Medically Underserved Area/
246. Rural Population/
247. Urban Population/
248. exp Ethnic Groups/
249. Minority Groups/
250. Vulnerable Populations/
251. ((health$ or social$ or racial$ or ethnic$) adj5 (inequalit$ or inequit$ or disparit$ or equit$ or disadvantage$ or depriv$)).tw.
252. (disadvant$ or marginali$ or underserved or under served or impoverish$ or minorit$ or racial$ or ethnic$).tw.
253. or/18-252
254. 17 and 253
255. 14 or 254
256. (femtosecond or phaco$ or keratometry or vitrectomy or endophthalmitis).ti.
257. (glaucoma$ or intraocular or IOL or keratoplast$ or refractive or retinopathy or tear or uveitis).ti.
258. or/256-257
259. 255 not 258
260. limit 259 to yr="2010 -Current"

For peer review only

Are we advancing Universal Health Coverage through cataract services? Protocol for a scoping review

Journal:	*BMJ Open*
Manuscript ID	bmjopen-2020-039458.R2
Article Type:	Protocol
Date Submitted by the Author:	02-Jun-2020
Complete List of Authors:	Lee, Chan; London School of Hygiene and Tropical Medicine, International Centre for Eye Health<br>Ramke, Jacqueline; LSHTM,<br>McCormick, Ian; London School of Hygiene and Tropical Medicine, Department of Clinical Research<br>Zhang, Justine; London School of Hygiene and Tropical Medicine Faculty of Infectious and Tropical Diseases, International Centre for Eye Health, Clinical Research Department<br>Aghaji, Ada; London Sch Hyg ,<br>Mwangi, Nyawira; London School of Hygiene and Tropical Medicine, ; Kenya Medical Training College,<br>Burn, Helen; Brighton and Sussex University Hospitals NHS Trust<br>Gordon, Iris<br>Yusufu, Mayinuer; Beijing Tongren Hospital; Beijing Institute of Ophthalmology<br>He, Mingguang; Sun Yat-sen University, State Key Laboratory of Ophthalmology, Zhongshan Ophthalmic Center; University of Melbourne, Centre for Eye Research Australia, Royal Victorian Eye and Ear Hospital<br>Silva, Juan Carlos; Pan American Health Organization, Life Course ; PAHO<br>Burton, Matthew J; London School of Hygiene and Tropical Medicine
**Primary Subject Heading**:	Ophthalmology
Secondary Subject Heading:	Epidemiology, Global health, Surgery, Health services research, Health informatics
Keywords:	Cataract and refractive surgery < OPHTHALMOLOGY, EPIDEMIOLOGY, PUBLIC HEALTH, Adult surgery < SURGERY, Ophthalmology < SURGERY

SCHOLARONE™
Manuscripts

I, the Submitting Author has the right to grant and does grant on behalf of all authors of the Work (as defined in the below author licence), an exclusive licence and/or a non-exclusive licence for contributions from authors who are: i) UK Crown employees; ii) where BMJ has agreed a CC-BY licence shall apply, and/or iii) in accordance with the terms applicable for US Federal Government officers or employees acting as part of their official duties; on a worldwide, perpetual, irrevocable, royalty-free basis to BMJ Publishing Group Ltd ("BMJ") its licensees and where the relevant Journal is co-owned by BMJ to the co-owners of the Journal, to publish the Work in this journal and any other BMJ products and to exploit all rights, as set out in our licence.

The Submitting Author accepts and understands that any supply made under these terms is made by BMJ to the Submitting Author unless you are acting as an employee on behalf of your employer or a postgraduate student of an affiliated institution which is paying any applicable article publishing charge ("APC") for Open Access articles. Where the Submitting Author wishes to make the Work available on an Open Access basis (and intends to pay the relevant APC), the terms of reuse of such Open Access shall be governed by a Creative Commons licence – details of these licences and which Creative Commons licence will apply to this Work are set out in our licence referred to above.

Other than as permitted in any relevant BMJ Author's Self Archiving Policies, I confirm this Work has not been accepted for publication elsewhere, is not being considered for publication elsewhere and does not duplicate material already published. I confirm all authors consent to publication of this Work and authorise the granting of this licence.

For peer review only

Are we advancing Universal Health Coverage through cataract services? Protocol for a scoping review

Authors:

Chan Ning Lee[1,2], Jacqueline Ramke[1,3], Ian McCormick[1], Justine H. Zhang[1,4], Ada Aghaji [1,5], Nyawira Mwangi[1,6], Helen Burn[1,7], Iris Gordon[1], Mayinuer Yusufu[8], Mingguang He[9], Juan Carlos Silva[10], Matthew J Burton[1,11]

Affiliations

1. International Centre for Eye Health, London School of Hygiene and Tropical Medicine, London, United Kingdom
2. St Paul's Eye Unit, Royal Liverpool University Hospital, Liverpool, United Kingdom
3. School of Optometry and Vision Science, University of Auckland, Auckland, New Zealand
4. Manchester Royal Eye Hospital, Manchester, United Kingdom
5. Department of Opthalmology, College of Medicine, University of Nigeria, Enugu, Nigeria
6. Department of Clinical Medicine, Kenya Medical Training College, Nairobi, Kenya
7. Beijing Institute of Ophthalmology, Beijing Tongren Eye Center, Beijing Tongren Hospital, Capital Medical University, Beijing, China
8. Department of Ophthalmology, Stoke Mandeville Hospital, Aylesbury, United Kingdom
9. Centre for Eye Research Australia, University of Melbourne, Royal Victorian Eye and Ear Hospital, East Melbourne, Australia
10. Division of Blindness Prevention, Pan American Health Organization, Bogota, Colombia
11. Moorfields Eye Hospital, London, United Kingdom

For peer review only - http://bmjopen.bmj.com/site/about/guidelines.xhtml

ABSTRACT

Introduction

Universal Health Coverage (UHC) includes the dimensions of equity in access, quality services that improve health, and protection against financial hardship. Cataract continues to be the leading cause of blindness globally, despite cataract surgery being an efficacious intervention. The aim of this scoping review is to map the nature, extent and global distribution of data on cataract services for UHC in terms of equity, access, quality and financial protection.

Methods and analysis

The search will be constructed by an Information Specialist and undertaken in MEDLINE, Embase and Global Health databases. We will include all published non-interventional primary research studies and systematic reviews that report a quantitative assessment of access, equity, quality or financial protection of cataract surgical services for adults at the sub-national, national, regional, or global level from population-based surveys or routinely collected health service data since 1 January 2000 and published through to February 2020.

Screening and data charting will be undertaken using Covidence systematic review software. Titles and abstracts of identified studies will be screened by two authors independently. Full text articles of potentially relevant studies will be obtained and reviewed independently by two authors against the inclusion criteria. Any discrepancies between the authors will be resolved by discussion, with a third author as necessary. A data charting form will be developed and piloted on three studies by three authors, and amendments made as necessary. Data will be extracted by two reviewers independently and summarised narratively and using maps.

Ethics and dissemination

Ethical approval was not sought as the scoping review will only use published and publicly accessible data. The review will be published in an open access peer-reviewed journal. A summary of the results will be developed for website posting, stakeholder meetings, and inclusion in the ongoing *Lancet Global Health* Commission on Global Eye Health.

For peer review only

STRENGTHS AND LIMITATIONS

- The broad scope of this review will result in the first synthesis to date of data on the UHC dimensions of cataract surgical services.
- Another strength is that we will include studies from all world regions and high-, low- and middle-income countries with no language restrictions, to give a global picture of cataract services.
- A potential limitation is the paucity of available information on the 'financial protection' dimension.

For peer review only

For peer review only - http://bmjopen.bmj.com/site/about/guidelines.xhtml

INTRODUCTION

Eye health and vision impairment represent a major global health concern. In the recent *World Report on Vision,* the World Health Organization (WHO) outlined how the provision of quality eye care services contributes directly to achieving Universal Health Coverage (UHC).[1] WHO estimated that in 2020 up to 2.2 billion people have some form of vision impairment and that this figure is projected to rise leading to an increased burden on health systems.[1]

Monitoring progress towards UHC

The WHO defines UHC in the following terms:

"Universal health coverage (UHC) means that all people and communities can use the promotive, preventive, curative, rehabilitative and palliative health services they need, of sufficient quality to be effective, while also ensuring that the use of these services does not expose the user to financial hardship".[2]

It has three broad principles: (1) equity in access; (2) quality services that improve health; (3) protection against financial risk. WHO and the World Bank have developed a framework for tracking progress towards UHC.[3] This focuses on two elements:

1. measuring the coverage of essential health services, as a proportion of the population that can access essential quality health services; and
2. measuring financial protection by determining the proportion of the population in whom direct payment made to obtain health services leads to financial hardship and/or a threat to living standards.[3]

A variety of different types of indicators are used. *Effective service coverage indicators* measure the proportion of people in need of services who receive services of sufficient quality to obtain potential health gains; these are preferred if available. *Service coverage indicators* measure the proportion of the population that needs a service that receive it. *Proxy indicators* are sometimes used where service coverage indicators are not available, but provide a correlated indication of the provision of a health service.[3 4]

The WHO and World Bank have selected a panel of 16 "tracer indicators" to monitor progress towards UHC.[2] There is currently no eye health related indicator among this panel though *effective cataract surgical coverage* and *effective refractive error coverage* were proposed in WHO's Thirteenth General Programme of Work 2019 – 2023 Impact Framework.[5] In addition to measuring population level coverage, it is very important to measure equity in service provision, by disaggregating the data and comparing sub-populations such as wealth quintiles, education, sex, age and geographical region.[3]

For peer review only - http://bmjopen.bmj.com/site/about/guidelines.xhtml

Monitoring cataract services for UHC

Cataract is the leading cause of blindness globally and is the second leading cause of vision impairment.[6] The last three decades have seen a marked increase in available data on vision impairment due to cataract, as well as cataract services. These data have enabled calculation of indicators of *access* and *quality* of cataract surgery, including:

- Cataract Surgical Rate (CSR): the number of cataract operations per million population per year.[7]
- Cataract Surgical Coverage (CSC): the number of people in a population who have received cataract surgery as a proportion of those having operable and operated cataract.[6]
- Cataract Surgical Outcome (CSO): the presenting visual acuity of the operated eye.
- Effective cataract surgical coverage (eCSC): the number of people in a population with operated cataract and a visual acuity of 6/18 or better as a proportion of those having operable and operated cataract.[8]

Effective Cataract Surgical Coverage (eCSC), has the characteristics of an *Effective service coverage indicator,* as preferred by the WHO/World Bank, as it combines information on the proportion of the population covered and the outcome of the surgical intervention.[9 10]

Disaggregation of larger datasets has allowed analyses of *equity* in cataract surgery as well, for example in highlighting existing gender disparities in CSC.[11-15] Much less data are available quantifying financial aspects of cataract services. To our knowledge, no existing synthesis of the distribution and quantity of known evidence for the UHC dimensions of cataract surgery has been undertaken. The aim of this scoping review is to map the nature, extent and global distribution of data on cataract surgical services for UHC in terms of equity, access, quality and financial protection.

We chose to undertake a scoping review rather than an alternative evidence synthesis approach because we wished to identify and map the available evidence, which we anticipate will be heterogeneous.[16]

METHODS

Objectives / Scoping review questions

We aim to answer the following two questions in relation to cataract services for UHC:

1. What is the nature, extent and global distribution of data on the coverage and effectiveness of cataract services?
2. What is the nature, extent and global distribution of data on financial protection in relation to cataract services?

For peer review only - http://bmjopen.bmj.com/site/about/guidelines.xhtml

Protocol and registration

The protocol for this scoping review is reported according to the relevant sections of the PRISMA Extension for Scoping Reviews (PRISMA-ScR) guideline (Annex 1).[17] The protocol is registered on the Open Science Framework (https://osf.io/k3mwg/).

Eligibility Criteria

We will include all published prospective and retrospective primary research studies and systematic reviews that report a quantitative assessment of access, equity, quality or financial protection of cataract surgical services for adults at the sub-national, national, regional, or global level (examples outlined in Table 1). We will include population-level observational studies and reports, including those that use routinely collected data (such as in health information systems) and household surveys such as Rapid Assessment of Avoidable Blindness (RAAB) surveys. We will exclude intervention studies and studies within clinical sub-populations as their outcomes can be different to the general population (e.g. people with diabetes, people with age related macular degeneration). We will exclude studies focused exclusively on cataract services for children (aged under 18 years), as these services differ substantially from those for age-related cataract.

To assess *access* we will include studies that report cataract surgical coverage and cataract surgical rate, which are priority indicators for monitoring global eye health.[18] Beyond these, we will include studies that report the number and distribution of human resources and surgical facilities. We acknowledge that there are many quantitative and qualitative elements of health care *quality* as defined by the WHO.[19] However, for the purposes of this review we will focus on only three—effective cataract surgical coverage, vision outcomes of cataract surgery and reported complications. We anticipate the literature reporting *financial protection* associated with cataract surgery will be limited. We will include studies and surveys that report 'catastrophic' and 'impoverishing' spending on cataract surgery according to WHO definitions, as well as other related measures of personal and government expenditure on cataract surgery and service provision (Table 1). We will use the PROGRESS acronym[20] to assess *equity*.

Studies will be limited to those including data collected since 1 January 2000 to provide a contemporary view of cataract services. The search strategy will be undertaken without language restrictions and translation will be arranged when required.

For peer review only - http://bmjopen.bmj.com/site/about/guidelines.xhtml

Table 1: Primary and secondary outcomes included in the review, mapped against UHC dimensions

UHC Dimension	Primary Cataract Indicator	Secondary Cataract Indicator
**Access (Coverage)** - *the availability of good health services within reasonable reach and available at the point of need.*	Cataract surgical coverage	• Cataract surgical rate[21]<br>• Number of operating surgeons by country<br>• Number and distribution of operating centres by country
**Quality** – *limited to the WHO quality elements of effectiveness and safety*	Effective cataract surgical coverage[8]	• Cataract Surgical Outcome (CSO)[11]<br>• Complication Rates per surgeon/institution
**Financial Protection** - *direct payments made to obtain health services do not expose people to financial hardship and do not threaten living standards.*	Rate of Catastrophic Spending on cataract surgery (25% of total household expenditure per WHO)<br>Rate of Impoverishing Spending on cataract surgery (as defined by income PPP/day/capita below country poverty line AND/OR international poverty line)	Cost of Cataract Surgery (to patient / household)[22,23]
**Equity** – *services are available for all who need them*	Disaggregation of any of the primary or secondary indicators by sex/gender[13,15,24]	Disaggregation of any of the primary or secondary indicators by any other PROGRESS factor[20]:<br>**P**lace of residence (e.g. urban/rural, sub-national unit)<br>**R**ace / ethnicity / culture / language<br>**O**ccupation<br>[**G**ender/sex]<br>**R**eligion<br>**E**ducation<br>**S**ocioeconomic status[12]<br>**S**ocial capital (e.g. marital status)[25]

Search Strategy

We will search Embase, MEDLINE and Global Health databases for studies published from 1 January 2000 through to February 2020 using search strategies developed by an Information Specialist from Cochrane Eyes and Vision (IG) (MEDLINE Search Strategy included in Annex 2). We will provide a list of included studies and reports to field experts and request they identify additional sources of both published and unpublished reports for consideration in the review.

We will use the RAAB repository (http://raabdata.info/) to identify all reports and data from sub-national and national RAAB studies taken from January 1, 2000 onwards.

To identify government and non-government reports in the grey literature, we will use a checklist adapted from the Canadian Agency for Drugs and Technologies in Health (CADTH) Grey Matters checklist to undertake a search of relevant websites.[26]

The following grey literature databases and repositories will be searched:

- OpenGrey (http://www.opengrey.eu/)
- Global Burden of Disease (http://www.healthdata.org/gbd)
- Global Health Data Exchange (http://ghdx.healthdata.org/)
- WHO (https://www.who.int/library/en/)

For peer review only - http://bmjopen.bmj.com/site/about/guidelines.xhtml

- IAPB (https://www.iapb.org/global-vision-database-maps/)
- National Ministry of Health websites.

Selection of Sources of Evidence

All titles and abstracts will be screened by at least two investigators independently using Covidence systematic review software (Veritas Health Innovation, Melbourne, Australia; available at www.covidence.org). Assessment of eligibility for inclusion will be carried out by two investigators independently with a third investigator reviewing discrepancies. Reference lists of all included articles will be examined to identify further potentially relevant reports. The study selection process will be summarised in a PRISMA flow diagram.

Data Charting

The data charting form will be developed in Covidence and piloted by investigators prior to use. Data charting will be carried out by two investigators independently. As data sources are expected to be heterogenous and broad in nature, data charting will be an iterative process throughout the review. Information that is absent or unclear will be addressed by contacting study authors with up to three attempts by email. The result of these attempts will be reported.

Because our focus is on mapping the availability of evidence, we did not to undertake quality appraisal of individual studies.[17]

Data Items

Source Characteristics

- Published Data Characteristics - Author(s), Year of Publication, Journal, Language,
- Grey Literature Characteristics - Author (Organisation e.g. WHO, Ministry of Health), Year of Publication, Source Website (e.g. government/non-government organisation), Language, Type of Literature (Report, Thesis, Technical Report, Statistics, other)

Study characteristics - Type of Study, Countries / regions investigated, Level of analysis (sub-national, national, regional, global), Sample details (frame, size), Year of data collection, Outcome(s) reported (as outlined in Table 1), UHC dimension(s) investigated (Access, Equity, Quality, Financial Protection).

Synthesis of Results

Following data charting, we will undertake narrative synthesis. Where possible maps will be used to summarise the available global, regional, national and sub-national distribution and proportion of studies reporting each UHC dimension for cataract surgery. Tables will be constructed to demonstrate

For peer review only - http://bmjopen.bmj.com/site/about/guidelines.xhtml

distribution of studies by region and (if appropriate) country. Where enough data are identified, further quantitative analyses of primary or secondary outcomes may be undertaken as a subsequent analysis.

Patient and Public Involvement

This protocol was developed with input from the Commissioners of the *Lancet Global Health* Commission on Global Eye Health,[27] which includes people with lived experience of vision impairment (and cataract surgery), policy makers, academics, clinicians, government eye health programme leaders and advocacy specialists.

Ethics and Dissemination

As this scoping review will only consider publicly available literature and reports, no ethics approval is required. Findings will be published in an open-access peer-reviewed journal, and a summary will be developed for website access and stakeholder meetings. A summary of the findings will also be included in the ongoing *Lancet Global Health* Commission on Global Eye Health.[27]

For peer review only

REFERENCES

1. World Health Organization. World Report on Vision. Geneva: WHO, 2019.
2. World Health Organization. What is universal coverage? [Accessed 1 March 2020]. Available from: http://www.who.int/health_financing/universal_coverage_definition/en/.
3. World Health Organization and The World Bank. Tracking universal health coverage: 2017 global monitoring report. Geneva: WHO, 2017.
4. Hogan DR, Stevens GA, Hosseinpoor AR, et al. Monitoring universal health coverage within the Sustainable Development Goals: development and baseline data for an index of essential health services. *The Lancet Global Health* 2018;6(2):e152-e68.
5. World Health Organization. 13th General Programme of Work (GPW13) WHO Impact Framework [Accessed 1 March 2020]. Available from: https://www.who.int/about/what-we-do/GPW13_WHO_Impact_Framework_Indicator_Metadata.pdf.
6. Flaxman S, Bourne R, Resnikoff S, et al. Global Causes of Distance Vision Loss: 1990-2015 and projections to 2020. *Lancet Global Health* 2017;5(12):e1221-e34.
7. Foster A. Cataract and "Vision 2020—the right to sight" initiative. *British Journal of Ophthalmology* 2001;85(6):635-37.
8. Ramke J, Gilbert C, Lee ACL, et al. Effective cataract surgical coverage: an indicator for measuring quality-of-care in the context of Universal Health Coverage *PLoS One* 2017;12(3):e0172342.
9. Ng M, Fullman N, Dieleman JL, et al. Effective coverage: a metric for monitoring universal health coverage. *PLoS Medicine* 2014;11(9):e1001730.
10. World Health Organization. World health statistics 2018: monitoring health for the SDGs, Sustainable Development Goals. Geneva: WHO, 2018.
11. Vijaya L, George R, Rashima A, et al. Outcomes of cataract surgery in a rural and urban south Indian population. *Indian Journal of Ophthalmology* 2010;58(3):223.
12. Yan W, Wang W, van Wijngaarden P, et al. Longitudinal changes in global cataract surgery rate inequality and associations with socioeconomic indices. *Clinical & Experimental Ophthalmology* 2019;47(4):453-60.
13. Prasad M, Malhotra S, Kalaivani M, et al. Gender differences in blindness, cataract blindness and cataract surgical coverage in India: a systematic review and meta-analysis. *British Journal of Ophthalmology* 2020;104(2):220-24.
14. Zhao J, Xu X, Ellwein LB, et al. Cataract Surgical Coverage And Visual Acuity Outcomes In Rural China In 2014 And Comparisons With The 2006 China Nine-Province Survey. *American Journal of Ophthalmology* 2018;193:62-70.
15. Lewallen S, Mousa A, Bassett K, et al. Cataract surgical coverage remains lower in women. *British Journal of Ophthalmology* 2009;93(3):295-98.
16. Munn Z, Peters MD, Stern C, et al. Systematic review or scoping review? Guidance for authors when choosing between a systematic or scoping review approach. *BMC Medical Research Methodology* 2018;18(1):143.
17. Tricco AC, Lillie E, Zarin W, et al. PRISMA extension for scoping reviews (PRISMA-ScR): checklist and explanation. *Annals of Internal Medicine* 2018;169(7):467-73.
18. World Health Organization. Universal Eye Health: A global action plan 2014-2019. Geneva: WHO, 2013.
19. World Health Organization. Delivering quality health services: a global imperative for universal health coverage. Geneva: WHO, 2018.
20. O'Neill J, Tabish H, Welch V, et al. Applying an equity lens to interventions: using PROGRESS ensures consideration of socially stratifying factors to illuminate inequities in health. *Journal of Clinical Epidemiology* 2014;67(1):56-64.
21. Wang W, Yan W, Müller A, et al. A global view on output and outcomes of cataract surgery with national indices of socioeconomic development. *Investigative Ophthalmology & Visual Science* 2017;58(9):3669-76.
22. Ibrahim N, Pozo-Martin F, Gilbert C. Direct non-medical costs double the total direct costs to patients undergoing cataract surgery in Zamfara state, Northern Nigeria: a case series. *BMC Health Services Research* 2015;15(1):163.

For peer review only - http://bmjopen.bmj.com/site/about/guidelines.xhtml

23. Fang J, Wang X, Lin Z, et al. Variation of cataract surgery costs in four different graded providers of China. *BMC Public Health* 2010;10(1):543.
24. Carter MJ, Limburg H, Lansingh VC, et al. Do gender inequities exist in cataract surgical coverage? Meta-analysis in Latin America. *Clinical & Experimental Ophthalmology* 2012;40(5):458-66.
25. Ramke J, Kyari F, Mwangi N, et al. Cataract Services are Leaving Widows Behind: Examples from National Cross-Sectional Surveys in Nigeria and Sri Lanka. *International Journal of Environmental Research and Public Health* 2019;16(20):3854.
26. Canadian Agency for Drugs and Technologies in Health. Grey matters: a practical tool for searching health-related grey literature Ottawa2018 [https://www.cadth.ca/resources/finding-evidence.]. Available from: https://www.who.int/about/what-we-do/GPW13_WHO_Impact_Framework_Indicator_Metadata.pdf.
27. Burton MJ, Faal HB, Ramke J, et al. Announcing The Lancet Global Health Commission on Global Eye Health. *The Lancet Global Health* 2019;7:e1612-13.

For peer review only

For peer review only - http://bmjopen.bmj.com/site/about/guidelines.xhtml

Authors' Contributions: JR and MJB conceived the idea for the review. CNL, JR and MJB drafted and revised the protocol with suggestions from IM, JHZ, AA, NM, HB, IG, MY, MH and JCS. IG constructed the search.

Corresponding Author: Jacqueline Ramke (Jacqueline.Ramke@lshtm.ac.uk)

Funding Statement: MJB is supported by the Wellcome Trust (207472/Z/17/Z). JR is a Commonwealth Rutherford Fellow, funded by the UK government through the Commonwealth Scholarship Commission in the UK. The Lancet Global Health Commission on Global Eye Health is supported by The Queen Elizabeth Diamond Jubilee Trust, Moorfields Eye Charity [grant number GR001061], NIHR Moorfields Biomedical Research Centre, Wellcome Trust, Sightsavers, The Fred Hollows Foundation, The SEVA Foundation, British Council for the Prevention of Blindness and Christian Blind Mission.

Competing Interests: None declared

Number of Tables: 1

Number of Figures: 0

Data Sharing Statement: Data generated from this review will be available upon reasonable request from Jacqueline.Ramke@lshtm.ac.uk

Keywords: cataract services, Universal Health Coverage, access, quality, financial protection, equity

Word Count: 1686

For peer review only - http://bmjopen.bmj.com/site/about/guidelines.xhtml

Annex 1: PRISMA SCR summary

SECTION	ITEM	PRISMA-ScR CHECKLIST ITEM	REPORTED ON PAGE #
TITLE			
Title	1	Identify the report as a scoping review.	1
ABSTRACT			
Structured summary	2	Provide a structured summary that includes (as applicable): background, objectives, eligibility criteria, sources of evidence, charting methods, results, and conclusions that relate to the review questions and objectives.	2
INTRODUCTION			
Rationale	3	Describe the rationale for the review in the context of what is already known. Explain why the review questions/objectives lend themselves to a scoping review approach.	4-6
Objectives	4	Provide an explicit statement of the questions and objectives being addressed with reference to their key elements (e.g., population or participants, concepts, and context) or other relevant key elements used to conceptualize the review questions and/or objectives.	6
METHODS			
Protocol and registration	5	Indicate whether a review protocol exists; state if and where it can be accessed (e.g., a Web address); and if available, provide registration information, including the registration number.	6
Eligibility criteria	6	Specify characteristics of the sources of evidence used as eligibility criteria (e.g., years considered, language, and publication status), and provide a rationale.	6-7
Information sources	7	Describe all information sources in the search (e.g., databases with dates of coverage and contact with authors to identify additional sources), as well as the date the most recent search was executed.	8
Search	8	Present the full electronic search strategy for at least 1 database, including any limits used, such that it could be repeated.	8
Selection of sources of evidence	9	State the process for selecting sources of evidence (i.e., screening and eligibility) included in the scoping review.	8
Data charting process	10	Describe the methods of charting data from the included sources of evidence (e.g., calibrated forms or forms that have been tested by the team before their use, and whether data charting was done independently or in duplicate) and any processes for obtaining and confirming data from investigators.	8-9
Data items	11	List and define all variables for which data were sought and any assumptions and simplifications made.	9
Critical appraisal of individual sources of evidence	12	If done, provide a rationale for conducting a critical appraisal of included sources of evidence; describe the methods used and how this information was used in any data synthesis (if appropriate).	na
Synthesis of results	13	Describe the methods of handling and summarizing the data that were charted.	9
FUNDING			
Funding	22	Describe sources of funding for the included sources of evidence, as well as sources of funding for the scoping review. Describe the role of the funders of the scoping review.	12

From: Tricco AC, et al.[18]

For peer review only

For peer review only

Annex 2: Search Results

MEDLINE

1. (cataract$ adj2 surg$ adj2 rate$).tw.
2. (rate$ adj2 cataract$ adj2 surg$).tw.
3. (cataract$ adj2 surg$ adj2 coverage$).tw. 4. (cataract$ adj2 surg$ adj2 outcome$).tw. 5. (incidence adj4 cataract$ adj2 surg$).tw. 6. (rapid adj2 assessment adj3 cataract$).tw. 7. Rapid Assessment of Avoidable Blindness.tw.
8. RAAB.tw.
9. Cataract Extraction/ec [Economics]
10. or/1-9
11. (universal adj2 health adj2 coverag$).tw.
12. cataract$.tw.
13. 11 and 12
14. 10 or 13
15. (cataract$ adj10 (district$ or region$ or province$ or state or states or territor$ or sub-national or national or nation$)).tw.
16. (cataract$ adj10 (country or countries or worldwide or global$ or Asia or China or India or Africa or Europe$)).tw.
17. 15 or 16
18. "Quality of Health Care"/
19. Quality Improvement/
20. Delivery of Health Care/
21. National Health Programs/ 22. State Medicine/
23. Regional Health Planning/ 24. Health Planning/
25. Health Plan Implementation/
26. Health Planning Guidelines/
27. Health Care Reform/
28. Health Resources/
29. Health Priorities/
30. Health Services Research/
31. "health services needs and demand"/
32. Needs Assessment/
33. State Health Plans/
34. Regional Health Planning/
35. Community Health Planning/
36. Hospital Planning/
37. Regional Medical Programs/
38. Health Maintenance Organizations/
39. Comprehensive Health Care/
40. Health Facility Planning/
41. Health Facility Administration/
42. Hospital Administration/
43. exp Hospitals, public/ 44. exp Hospitals, private/
45. health system$.tw.
46. Models, Organizational/
47. Decision Making, Organizational/ 48. Resource Allocation/
49. Efficiency, Organizational/
50. Organizational Innovation/
51. Delivery of Health Care, Integrated/
52. Interdisciplinary Communication/
53. Public Health/
54. Health Promotion/
55. Policy Making/
56. Program Development/
57. Program Evaluation/
58. Quality Control/
59. Quality Assurance, Health Care/
60. Benchmarking/
61. Capacity Building/
62. Health Services Accessibility/ 63. Health Policy/
64. Surgical Procedures, Operative/
65. exp Surgical Equipment/
66. Health Care Rationing/
67. Medically Underserved Area/ 68. Healthcare Disparities/
69. Health Status Disparities/
70. exp Attitude to Health/
71. "Patient Acceptance of Health Care"/
72. Health Education/
73. Public Opinion/

For peer review only

74. Health Behavior/ 77. exp Communication/

75. Social Behavior/ 78. exp Culture/

76. Superstitions/

79. Sex Factors/ 122. quality management.tw.

80. (cataract$ adj3 (woman or women or female or gender or 123. (organisation$ adj3 cultur$).tw.

sex or sexual or equit$ or inequit$)).tw. 124. Disease Management/

81. Women's Rights/ 125. Program Evaluation/

82. Prejudice/ 126. ((provider$ or program$) adj3 (monitor$ or evaluate$ or

83. Vulnerable Populations/ modif$ or practice)).tw.

84. Social Responsibility/ 127. (implement$ adj3 (improve$ or change$ or effort$ or

85. Social Welfare/ issue$ or impede$ or glossary or tool$ or innovation$ or 86. Urban Health Services/ outcome$ or driv$ or examin$ or reexamin$ or scale$ or

87. Rural Health Services/ strateg$ or advis$ or expert$)).tw.

88. Rural Population/ 128. (needs adj3 assess$).tw.

89. Patient Escort Service/ 129. ((education$ or learn$) adj5 (continu$ or material$ or

90. Health Manpower/ meeting or collaborat$)).tw.

91. Health Personnel/ 130. exp Medical audit/

92. Health Workforce/ 131. (audit or feedback or compliance or adherence or

93. Workforce/ training or innovation).ti.

94. human resources for eye health.tw. 132. (guideline$ adj3 (clinical or practice or implement$ or

95. HReH.tw. promot$)).tw.

96. Medical Staff, Hospital/ 133. exp Health Services Accessibility/

97. Nursing Staff, Hospital/ 134. (outreach adj2 (service$ or visit$)).tw.

98. Personnel, Hospital/ 135. (intervention$ adj3 (no or usual or routine or target$ or

99. Professional Competence/ tailor$ or mediat$)).tw. 100. Clinical Competence/ 136. usual care.tw. 101. Medical Errors/ 137. exp Reminder Systems/ 102. Clinical Governance/ 138. remind$.tw.

103. Government Regulation/ 139. (improve$ adj3 (attend$ or visit$ or intervention$ or

104. Public Policy/ adhere$)).tw.

105. Public Health Practice/ 140. (increas$ adj3 (attend$ or visit$ or intervention$ or 106. Public Health Administration/ adhere$)).tw.

107. Health Plan Implementation/ 141. (appointment$ adj3 (miss$ or fail$ or remind$ or follow 108. Public-Private Sector Partnerships/ up)).tw. 109. Delivery of Health Care, Integrated/ 142. Telephone/ 110. service delivery.tw. 143. telephone.tw.

111. decision making.tw. 144. Cell Phones/

112. (consensus adj3 (process$ or discuss)).tw. 145. Mobile Applications/

113. stakeholder$.tw. 146. Remote Consultation/

114. Quality Control/ 147. (m-health or e-health or g-health or u-health).tw.

For peer review only

115. Total Quality Management/ 148. (phone$ adj1 (smart or cell)).tw. 116. Quality Indicators, Health Care/ 149. (smartphone$ or cellphone$).tw. 117. Quality Assurance, Health Care/ 150. (hand adj1 held device$).tw.

118. quality assurance.tw. 151. (mobile adj2 (health or healthcare or phone$ or device$ 119. (quality adj2 improv$).tw. or monitor$ or comput$ or app or apps or application)).tw.

120. total quality.tw. 152. Primary Health Care/

121. continuous quality.tw.

153. General Practitioners/ or Physicians, Family/ or Physicians, Primary Care/

154. Primary Prevention/

155. Preventive Health Services/

156. Community Health Services/

157. Community Health Nursing/

158. Health Services, Indigenous/

159. Rural Health Services/ 160. Mobile Health Units/

161. (Ophthalmologist$ or Optometrist$ or Optician$ or Orthopist$ or Refractionists).tw. 162. ((Ophthalmic or eye) adj3 (surgeon$ or nurse$ or technician$ or officer$ or assistant$ or staff$)).tw.

163. Physician's Practice Patterns/

164. Professional Practice/

165. (professional adj3 (practice or develop$ or educat)).tw.

166. Education, Medical, Continuing/

167. exp nurses/

168. Specialties, Nursing/

169. Nurse's Role/ 170. Education, Nursing, Continuing/ 171. (nurse or nurses).tw.

172. ((role or roles) adj3 expan$).tw.

173. (task$ adj3 shift$).tw.

174. exp Medical Records Systems, Computerized/

175. Management Information Systems/

176. Database Management Systems/

177. Computer Systems/ 178. Point-of-Care Systems/

179. Hospital Information Systems/

180. ((health or healthcare) adj4 (record or management system$)).tw.

181. (decision adj5 support).ti. 182. Economics/

183. "costs and cost analysis"/

184. Cost allocation/

185. Cost-benefit analysis/

186. Cost control/

187. Cost savings/

188. Cost of illness/

189. Cost sharing/

190. "deductibles and coinsurance"/ 191. Medical savings accounts/

192. Health care costs/

193. Direct service costs/

194. Drug costs/

195. Employer health costs/ 196. Hospital costs/ 197. Health expenditures/ 198. Capital expenditures/

199. Value of life/

200. exp economics, hospital/ 201. exp economics, medical/ 202. Economics, nursing/

203. Economics, pharmaceutical/

204. exp "fees and charges"/ 205. exp budgets/ 206. (low adj cost).mp. 207. (high adj cost).mp.

208. (health?care adj cost$).mp.

209. (fiscal or funding or financial or finance).tw.

210. (cost adj estimate$).mp.

211. (cost adj variable).mp. 212. (unit adj cost$).mp.

213. (economic$ or pharmacoeconomic$ or price$ or pricing).tw.

214. Uncompensated Care/

For peer review only

215. Reimbursement Mechanisms/ 216. Reimbursement, Incentive/
217. (insurance adj3 (health$ or scheme$)).tw.
218. (financial or economic or pay or payment or copayment or paid or fee or fees or monetary or money or cash or incentiv$ or disincentiv$).tw.
219. ((pay or paying or paid or cost$ or free or wait$ or qualit$) adj3 surg$).tw. 220. (will$ adj3 pay$).tw. 221. (waiting adj2 time).tw.
222. ((surgery or surgical or surgeon$ or ophthalmologist$) adj2 (experience$ or supervis$ or rate or rates or output or volume or uptake number$ or coverage or annual$)).tw.
223. Gross Domestic Product/
224. Medicare/
225. human development index.tw.
226. gross domestic product.tw.
227. (HDI or GDP).tw.
228. Cataract Extraction/sn [Statistics & Numerical Data] 229. (global adj2 burden adj2 cataract$).tw.
230. (cataract$ adj3 (cost$ or income$ or price$ or reimburse$)).tw.
231. exp Patient Acceptance of health Care/
232. exp Attitude to Health/ 233. exp Health Behavior/ 234. Health Education/
235. exp Patient Education as Topic/
236. exp Health Promotion/ 237. Socioeconomic Factors/
238. exp Poverty/ 239. Social Class/
240. Educational Status/
241. ((school or education$) adj3 (status or level$ or attain$ or achieve$)).tw. 242. Employment/
243. Healthcare Disparities/
244. Health Status Disparities/ 245. exp Medically Underserved Area/
246. Rural Population/
247. Urban Population/ 248. exp Ethnic Groups/ 249. Minority Groups/
250. Vulnerable Populations/
251. ((health$ or social$ or racial$ or ethnic$) adj5 (inequalit$ or inequit$ or disparit$ or equit$ or disadvantage$ or depriv$)).tw.
252. (disadvant$ or marginali$ or underserved or under served or impoverish$ or minorit$ or racial$ or ethnic$).tw.
253. or/18-252 254. 17 and 253 255. 14 or 254
256. (femtosecond or phaco$ or keratometry or vitrectomy or endophthalmitis).ti.
257. (glaucoma$ or intraocular or IOL or keratoplast$ or refractive or retinopathy or tear or uveitis).ti.
258. or/256-257 259. 255 not 258
260. limit 259 to yr="2010 -Current"

For peer review only
